# Chronic Psychological Stress Induces Cardiomyocyte Hypertrophy Through Corticosterone‐Glucocorticoid Receptor‐LAMA5 Axis

**DOI:** 10.1002/advs.202414659

**Published:** 2025-07-11

**Authors:** Chuanjing Zhang, Yongfei Song, Xiaojun Jin, Qingbo Xu, Honghua Ye, Zhuonan Wu, Hui Lin, Jiale Hu, Chen Huang, Jianqing Zhou, Jiangfang Lian

**Affiliations:** ^1^ The Affiliated Lihuili Hospital of Ningbo University Health Science Center Ningbo University Ningbo Zhejiang 315000 China; ^2^ Department of Cardiology The First Affiliated Hospital Zhejiang University School of Medicine Hangzhou Zhejiang 310003 China; ^3^ Department of Cardiology Shaoxing Second Hospital Shaoxing Zhejiang 312000 China; ^4^ Department of Cell Biology and Genetics School of Basic Medical Sciences Xi'an Jiaotong University School of Health Science Center Xi'an Shaanxi 710061 China; ^5^ Key Laboratory of Environmentally and Genetically Associated Diseases Xi'an Jiaotong University School of Health Science Center Xi'an Shaanxi 710061 China

**Keywords:** cardiomyocyte hypertrophy, chronic psychological stress, corticosterone, glucocorticoid receptors, laminin subunit alpha 5

## Abstract

Chronic psychological stress is closely related to myocardial diseases, but the pathological processes and related mechanisms are not yet clear. In this study, 5 batches of animal experiments are conducted to reveal the pathological process of chronic psychological stress on the heart, and found that cardiomyocyte hypertrophy is an early manifestation of cardiac damage. Furthermore, it is discovered that corticosterone and glucocorticoid receptor (GR) play an important role in mediating this phenomenon. Additionally, a new direct target gene of GR, *Lama5*, is identified and confirmed in vivo and in vitro that the corticosterone‐GR‐LAMA5 axis is a significant pathway mediating chronic psychological stress‐induced cardiomyocyte hypertrophy. Moreover, the potential of LAMA5 is validated as a biomarker for myocardial hypertrophy and impaired cardiac function in patients with depression. In summary, a novel hypertrophy‐related gene, *Lama5* has been identified, that may play an important role in chronic psychological stress‐induced cardiomyocyte hypertrophy.

## Graphical abstract

In vivo experiments suggest that cardiomyocyte hypertrophy is an early manifestation of cardiac pathological changes induced by chronic psychological stress. In vivo and in vitro experiments suggest that the corticosterone‐glucocorticoid receptor‐LAMA5 axis mediates cardiomyocyte hypertrophy induced by chronic psychological stress. The clinical results suggest that LAMA5 is a potential specific marker of heart failure in depressed patients.

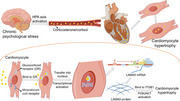

## 1 Introduction

Psychological stress exerts a significant impact on cardiovascular disease.^[^ ^1^ ^]^ For instance, it is well established that acute psychological stress can precipitate acute coronary syndrome^[^ ^2^, ^3^ ^]^ or stress cardiomyopathy.^[^ ^4^ ^]^ Moreover, chronic psychological stress has been increasingly recognized as a contributing factor to hypertension^[^ ^5^ ^]^, atherosclerosis^[^ ^6^ ^]^, and other vascular‐related pathologies. However, the effects of chronic psychological stress on myocardial structure and function remain inadequately understood.

In clinical practice, prevalent diseases characterized by chronic psychological stress as a primary etiological factor or clinical manifestation include anxiety and depression, which have been proven to be independent risk factors for various cardiovascular diseases, notably heart failure.^[^ ^7^ ^]^ The prevalence of anxiety and depression among heart failure patients significantly exceeds that observed in the general population, and individuals with these comorbidities frequently experience poorer prognoses.^[^ ^8^, ^9^ ^]^ Nevertheless, the underlying pathological processes and mechanisms remain inadequately understood. Recent research has demonstrated that repeated exposure to social failure stress results in impaired cardiac muscle contraction performance and myocardial fibrosis in male rats.^[^ ^10^, ^11^ ^]^ The underlying mechanism is hypothesized to involve the activation of the sympathetic nervous system and the hypothalamic‐pituitary‐adrenal axis, triggered by chronic psychological stress. This activation leads to an increase in stress hormone levels, including adrenaline and glucocorticoids (predominantly cortisol in humans and corticosterone in rodents). Nonetheless, additional evidence is required to substantiate these findings comprehensively.

In this study, we conducted a comprehensive examination of the pathological processes in cardiac structure and function in rat models subjected to chronic psychological stress, utilizing five distinct batches. Our findings suggest that cardiomyocyte hypertrophy may serve as an early indicator of stress‐induced cardiac pathology. Subsequently, we conducted an analysis of transcriptomic alterations and serum hormone levels in heart tissues subjected to chronic psychological stress. This investigation led to the identification of a novel glucocorticoid receptor (GR) target gene, laminin subunit alpha 5 (*Lama5*), and an exploration of its association with cardiomyocyte hypertrophy. Our findings suggest that the corticosterone (CORT)‐GR‐LAMA5 axis may represent a potential mechanism and therapeutic target for cardiomyocyte hypertrophy induced by chronic psychological stress.

## 2 Experimental Section

### 2.1 Rats

For all animal studies, age‐matched male Wistar rats (purchased from Charles River) between 5 and 6 weeks of age were used. The rats were maintained in a specific pathogen‐free environment at a temperature of 22 °C, with a 12‐h light/dark cycle. The experimental protocol received approval from the Experimental Animal Ethics Committee of Ningbo University (batch number: 12654), and all procedures adhered to the guidelines for the care and use of experimental animals as stipulated by the National Institutes of Health of the United States.

### 2.2 Chronic Psychological Stress Model

Rats were randomly assigned to either a control group (n = 40) or a chronic mild stress (CMS) group (n = 40). CMS was a widely used depression animal model that simulates unpredictable, random adverse stimuli in daily human life. This study made slight modifications to the existing model methods.^[^ ^12^ ^]^ Following a 1‐week acclimation period, the rats in the CMS group were subjected to a 20‐week regimen of daily mild stressors, including: 18 h of food or water deprivation; 24 h of wet bedding (200 mL of water per 300 g of bedding); 24 h without bedding material; 24 h with a 45° cage tilt; 5 min of tail clipping; 24 h of inverted photoperiod (reversed day and night cycle); 36 h of continuous lighting; 30 min of cat meow stimulation (60dB) and bind for 1h. Each day, a random stressor was assigned, and the same stressor was applied to all rats in the experimental groups on that day. To enhance intra‐group comparability, the time difference in the onset of stress induction across all experimental groups was kept within 15 min. The daily stressor schedules for this study are documented in Table S1 (Supporting Information). The rats in the control group were fed under standard conditions without exposure to stress. Throughout the duration of the experiment, the weight and overall health of the rats were systematically monitored. The rats from both groups were subdivided into five batches, with one batch being euthanized every 4 weeks. The whole heart tissues were weighed and then stored in a refrigerator at −80 °C or fixed in 4% paraformaldehyde. The fifth batch of fresh left ventricular tissues was taken for full transcriptome sequencing.

### 2.3 Sucrose Preference Test (SPT)

Prior to the commencement of the experiment, a two‐day period of adaptive training was conducted to acclimate the rats to consuming a 1% sucrose solution. Sucrose preference tests were administered over a 24‐h period on the first day of the experiment and subsequently every four weeks. During these tests, two drinking bottles—one containing a 1% sucrose solution and the other containing plain water—were provided to measure the intake of both solutions over a 24‐h period. The sucrose preference index, defined as the ratio of sucrose solution intake to total water intake, was calculated to evaluate anhedonia in the rats.

### 2.4 Open Field Test (OFT)

Open field tests were administered at four‐week intervals following the initiation of CMS to evaluate behavioral alterations in the rats. The OFT was a widely recognized method for assessing depression‐like behaviors as well as autonomous behaviors in novel environments. The experimental apparatus consisted of an open field box measuring 100 cm × 100 cm × 40 cm. Prior to each trial, the rats were positioned at the center of the box and permitted to explore freely for a duration of 5 min. The movements of the rats were recorded via video surveillance, and the total distance traveled within the 5‐min period was quantified and analyzed using ANY‐Maze Software version 7.4 (Stoelting Co., USA).

### 2.5 Forced Swimming Test (FST)

A cylindrical glass tank with dimensions of 30 cm in diameter and 60 cm in height was utilized, into which water was added to a depth of 45 cm. The water temperature was standardized to 26 °C prior to the commencement of each experimental trial. Rats were individually introduced into the tank for a duration of 6 min, during which their movements were recorded using a video surveillance system. Following the 6‐min period, the rats were removed from the tank, placed on dry bedding, and warmed using an infrared lamp. The durations of swimming, struggling, and immobility behaviors in the water were subsequently analyzed and quantified using ANY‐Maze Software version 7.4 (Stoelting Co., USA). The videos were analyzed manually, and the analyst was unaware of the grouping of the rats.

### 2.6 Echocardiography

High‐frequency echocardiography (D6VET, Vinno) was employed at four‐week intervals to assess the cardiac function of the rats. The rats were anesthetized using 2% isoflurane and maintained on a heated mat to prevent hypothermia. Ultrasound gel was applied to the chest, and a high‐frequency probe (40 MHz) was utilized to obtain M‐mode and B‐mode images. Left ventricular ejection fraction (LVEF), fractional shortening (FS), diastolic interventricular septal (IVSd) thickness, and diastolic left ventricular posterior wall (LVPWd) thickness were recorded and analyzed.

### 2.7 Blood Pressure Measurement

The systolic blood pressure of the same cohort of rats was assessed at 4‐week intervals utilizing tail‐cuff plethysmography with the BP‐2010 Blood Pressure Analysis System. Measurements were conducted prior to the commencement of behavioral experiments, between 8:00 and 10:00 AM. The procedure was acclimatized over a one‐week period preceding the formal data collection.

### 2.8 Hematoxylin and Eosin (HE) and Masson Staining

The rat heart tissue was fixed in a 4% paraformaldehyde solution for 48 h, followed by paraffin embedding. Transverse sections were then cut at 5 µm intervals at the level of the left central ventricular papillary muscle. According to the experimental protocol, HE staining and Masson trichrome staining were performed using the HE staining kit (Solarbio) and the Masson trichrome staining kit (Solarbio), respectively. The morphology, thickness, and fibrosis of the myocardium were examined using a DMi1 Inverted Microscope (Leica). The proportion of the fibrotic area was quantified using ImageJ software.

### 2.9 Wheat Germ Agglutinin Staining

Heart sections were stained with AF488‐labeled wheat germ agglutinin (WGA, AAT Bioquest) to evaluate the cross‐sectional area of cardiomyocytes. The myocardial cell area was visualized and documented using a fluorescence microscope (DMi8, Leica) and subsequently analyzed with ImageJ software. Each rat was photographed in the middle left ventricle of 4 fields, each field containing 50–100 cells, and the mean of all cells was taken as the myocardial cell area of the rat. The area of cardiomyocytes was compared by nested analysis.

### 2.10 Enzyme Linked Immunosorbent Assay (ELISA)

A volume of 0.5 mL of blood was collected from the orbital sinus of the rats every 4 weeks. The samples were allowed to stand at room temperature for 1 h, followed by centrifugation at 2500 RPM for 15 min. The supernatant was then aliquoted and stored at −80 °C. According to the respective ELISA kit instructions, the serum from each batch of rats was analyzed for CORT (Elabscience), norepinephrine (Cusabio), serotonin (Cusabio), angiotensin II (Elabscience), aldosterone (Elabscience), and N‐terminal pro‐B‐type natriuretic peptide (NT‐proBNP, Elabscience).

### 2.11 Interventions of CORT‐GR‐LAMA5 In Vivo

Wistar rats were randomly assigned to five experimental groups: control, CMS, CMS+ metyrapone, CMS+ relacorilant, and CMS+ LAMA5KD, with each group comprising eight mice. Prior to the commencement of the experiment, rats of the CMS+ LAMA5KD group were administered 200 µL of AAV carrying *Lama5* shRNA via the tail vein at a viral titer of 2.93 × 10^13^ vg mL^−1^. The following LAMA5^−^ AAV was used, and detailed sequence information is available upon request: pcAAV‐cTnTo‐miR30shRNA (Lama5)‐WPRE (OBiO Technology). The serotype of the administered virus was MyoAAV1A. The virus utilized cardiac troponin T (cTnT) promoter to facilitate targeted aggregation of cardiomyocytes. Additionally, 200 µL of a non‐functional negative control virus was administered via tail vein injection to the remaining groups of rats. Following the initiation of the experiment, all rats, with the exception of the control group, were subjected to CMS stimulation for a duration of 12 weeks, employing the same stimulation protocol as previously described. Among them, the CMS+ metyrapone group was given metyrapone (MedChemExpress) 25mg kg^−1^ every 2 days, the CMS+ relacorilant group was given relacorilant (MedChemExpress) 30mg kg^−1^ every 2 days, and the other groups were given the same volume of saline at the same time. During the final week of the experiment, the OFT, SPT, and echocardiography were conducted. Upon completion of the experimental procedures, the rats were anesthetized and euthanized. Subsequently, the entire heart tissue was then excised, weighed, and preserved either in 4% paraformaldehyde or rapidly frozen in liquid nitrogen at −80 °C for future analysis.

### 2.12 H9C2 Cell Line Culture

H9C2 myocardial cell lines, procured from the Shanghai Cell Bank of the Chinese Academy of Sciences, were cultured in Dulbecco's Modified Eagle Medium (DMEM; Sigma–Aldrich) supplemented with 10% fetal bovine serum and 1% penicillin‐streptomycin. The cultures were maintained in a 5% CO_2_ incubator at 37 °C, with medium changes performed every 2–3 days to ensure appropriate cell density.

### 2.13 Cellular Drug Intervention

H9C2 cells were laid on 30% density plate, fresh medium was changed 24 h later, and CORT (1µmol L^−1^), relacorilant (1µmol L^−1^), finerenone (10µmol L^−1^), LY294002 (2µmol L^−1^) and other drugs were given according to the group intervention, The medium and drugs were refreshed every 24 h, with a total drug exposure duration of 48 h. For transient siRNA transfection, lipo3000 (Thermo Fisher Scientific) was utilized to transfect *GR*/*Lama5* siRNA according to the manufacturer's instructions. Subsequent drug treatments were administered as previously described. Scramble siRNA was used as the negative control; the specific sequences of the negative control siRNA and the siRNAs specific for *GR* and *Lama5* are provided in Table S2 (Supporting Information).

### 2.14 Establishment of Stable Cell Lines Overexpressing LAMA5

In this experiment, BrainVTA Technology Biological Co., Ltd. was commissioned to synthesize a LAMA5‐overexpressing lentivirus (pLV‐U6‐sgRNA(Lama5)‐EFS‐EGFP‐T2A‐Puro‐CMV‐SV40NLS‐dCas9‐NLS‐VPR‐WPRE, BrainVTA). Following the determination of the lethal concentration of puromycin in H9C2 cells, both the LAMA5‐overexpressing lentivirus and the control virus were transfected according to the standard procedures specified by the company. 24 h later, fresh medium was replaced and 1.5umol L^−1^ purinomycin was given (every 24 h) for 7 consecutive days of screening, and a high‐purity Lama5 overexpression stable strain was obtained.

### 2.15 Dual Luciferase Reporter Gene Assay

In this study, GenePharma was engaged to construct a GPL4 recombinant plasmid containing the *Lama5* promoter sequence and a pcDNA3.1 recombinant plasmid containing the full‐length GR sequence. These plasmids were subsequently transfected into H9C2 cells utilizing lipo3000 (Thermo Fisher Scientific). After a 24‐h incubation period, a dual luciferase assay (GenePharma) was conducted in accordance with the manufacturer's protocol. Luciferase activity from both firefly and renilla luciferases was quantified using a BioTek microplate reader to assess the regulatory effect of GR on *Lama5* promoter activity. The activity of firefly luciferase and renilla luciferase was quantified using a BioTek instrument, and the regulation of *Lama5* promoter activity by GR was assessed. The relative activity of firefly luciferase was calculated based on the activity of renilla luciferase.

### 2.16 Immunofluorescence

The cells were cultured in specialized cell culture dishes, fixed with 4% paraformaldehyde for 10 min, and subsequently permeabilized with 0.1% Triton X‐100 for 15 min. Tissue sections underwent standard dewaxing, rehydration, and antigen retrieval procedures, followed by blocking with phosphate‐buffered saline (PBS) containing 1% bovine serum albumin for 30 min. Primary antibodies were then added and incubated overnight at 4 °C. On the following day, the cells were incubated with fluorescently labeled secondary antibodies at a 1:400 dilution for 1 h, and the nuclei were counterstained with DAPI for 10 min. Fluorescence microscopy (Leica DMi8) was used to observe and photograph the localization and expression of target proteins in cells.

### 2.17 Western Blot (WB)

Total cellular or tissue proteins were extracted using RIPA lysis buffer supplemented with a protease/phosphatase inhibitor cocktail (Epizyme Biotech). Protein concentrations were quantified utilizing a BCA assay kit (Epizyme Biotech). Following SDS‐PAGE, the protein samples were transferred onto a PVDF membrane (Sigma–Aldrich). The membrane was then blocked with 5% skim milk powder in PBS‐Tween 20 buffer for 1 h and subsequently incubated overnight at 4 °C with specific primary antibodies. Primary antibodies used were as follows: anti‐MYH7 (1:1000, 22280‐1‐AP, Proteintech Group, Inc), anti‐CLO1A2, (1:5000, 66761‐1 ‐PBS, Proteintech Group, Inc), anti‐GR (1:5000, 24050‐1‐AP, Proteintech Group, Inc), anti‐Histone H3 (1:3000, 17168‐1‐AP, Proteintech Group, Inc), anti‐LAMA5 (1:1000, ab184330, Abcam), anti‐ Phospho‐PI3K (1:1000, #AF3242, Affinity Biosciences LTD.), anti‐PI3K (1:5000, 60225‐1‐Ig, Proteintech Group, Inc), anti‐ Phospho‐Akt (1:1000, #4060, Cell Signaling Technology, Inc.), anti‐AKT (1:2000, 10176‐2‐AP, Proteintech Group, Inc), anti‐ITGB1 (1:2000, 12594‐1‐AP, Proteintech Group, Inc), anti‐GAPDH (1:50000, 60004‐1‐Ig, Proteintech Group, Inc). The following day, an HRP‐labeled secondary antibody at a 1:500 dilution was incubated for 1 h. Color development was performed using an ECL kit from Epizyme Biotech. Protein bands were subsequently detected utilizing the ImageQuant LAS 500 chemiluminescence imaging system (Bio‐Rad) and analyzed with ImageJ software.

### 2.18 Quantitative Polymerase Chain Reaction (qPCR)

Total RNA was extracted from whole cells or tissues utilizing the TRIzol kit (Vazyme). Complementary DNA (cDNA) synthesis was performed using the reverse transcription kit (Vazyme), and amplification was conducted with the SYBR Green qPCR Master Mix (Vazyme). The target genes analyzed included *GR*, *Lama5*, and *Myh7*, among others. Relative gene expression levels were determined using the ΔΔCt method, with *Gapdh* serving as the reference gene. The primer sequences used in this study were as follows: rat *Myh7* (forward: 5′‐ATGGACCTGGAGAACGACA‐3′; reverse: 5′‐ GCGTGCCTGAAGCTCTTT‐3′), rat *Lama5* (forward: 5′‐CACAGCAACCACACCACA‐3′; reverse: 5′‐ TCAGACATCCTCGGTAGGC‐3′), rat *Gapdh* (forward: 5′‐ TCTCTGCTCCTCCCTGTTC‐3′; reverse: 5′‐ ACACCGACCTTCACCATCT‐3′).

### 2.19 Transcriptome Sequencing of Cardiac Tissue and H9C2 Cell

The transcriptome sequencing experiment described in this study was performed by Shanghai OE Biotechnology Co., Ltd. Tissue sequencing included the left ventricular wall tissues of the control group (n = 5) and the CMS group (n = 10) in week 21, weighing ≈200mg. Cell sequencing was divided into control group, CORT group, and CORT+R group, with 5 compound pores in each group, and the number of cells in each pore was greater than 1×10^6^. The following is a summary of the procedural steps: Initially, total RNA was extracted from the samples. Subsequently, ribosomal RNA was removed utilizing a ribo‐zero kit. The RNA was then fragmented into short segments through the application of a fragmentation reagent. These RNA fragments served as templates for the synthesis of the first strand of complementary DNA (cDNA) using six‐nucleotide random primers. Subsequently, a two‐strand complementary DNA (cDNA) synthesis reaction system was established to synthesize the second strand of cDNA. The purified cDNA strand underwent a series of preparatory steps, including end repair, addition of adenine (A) tails, and ligation of sequencing adapters. Subsequently, the fragments were size‐selected, followed by PCR amplification. Following quality control, genome comparison, and transcription splicing of the initial sequencing data, a quantitative analysis was conducted to identify differentially expressed genes. Subsequently, gene function and pathway enrichment analyses, including Gene Ontology (GO), Kyoto Encyclopedia of Genes and Genomes (KEGG)^[^ ^13^ ^]^, and Gene Set Enrichment Analysis (GSEA), were performed to investigate the impact of chronic psychological stress on cardiac gene expression.

### 2.20 Clinical Information and Plasma Sample Collection

Plasma samples were collected from healthy controls, depression (DP) patients with or without heart failure (HF), and HF patients without DP at Shaoxing Second Hospital. The clinical characteristics of the cohort are detailed in **Table** **1**. The protocol for the collection of human samples was approved by the Ethics Committee of Shaoxing Second Hospital (Ethics batch number: 2024052), and all participants provided written informed consent. Furthermore, ELISA kits (Elabsicence, China) were utilized to measure plasma cortisol and LAMA5 levels, following the manufacturer's protocols.

**Table 1:** Clinical information in our cohort.

Clinical variables	Control [n = 54]	DP without HF [n = 51]	DP with HF [n = 34]	HF without DP [n = 62]
Age (year)	60.60±8.25	63.12±8.12	65.97±8.69	65.19±6.35
Gender (male)	24 (44.44%)	22 (43.14%)	14 (41.18%)	29 (46.77%)
CHOL (mmol L^−1^)	4.20±0.81	4.18±1.10	3.85±1.36	4.40±1.01
LDL (mmol L^−1^)	2.85±0.80	2.64±0.69	3.09±0.65	3.22±0.90
Glu (mmol L^−1^)	4.95±0.63	5.14±0.89	5.52±1.21	5.72±1.49
BNP (pg mL^−1^)	47.96±36.95	44.82±27.88	164.1±113.1	373.7±217.8
cTnT (ng mL^−1^)	0.003±0.003	0.004±0.003	0.006±0.009	0.006±0.007
IVSd (mm)	9.36±1.17	9.26±1.10	10.57±1.42	10.52±1.49
LVPWd (mm)	9.07±1.29	9.11±0.96	10.25±1.32	10.41±1.35
LVEF (%)	67.51±6.95	68.20±8.63	53.13±11.32	49.20±9.89
FS (%)	39.91±5.56	40.52±4.69	36.15±4.24	30.30±6.47

### 2.21 Screening of Diagnostic Biomarkers of DP Combined with HF

Clinical parameters encompassing age, sex, history of underlying diseases, fasting blood glucose levels, B‐type natriuretic peptide (BNP), troponin, and ELISA results for cortisol and LAMA5 were collected. Least Absolute Shrinkage and Selection Operator (LASSO) regression analysis was employed to identify diagnostic markers for HF in patients with depression. This analysis ultimately identified two significant indicators: BNP and LAMA5.

### 2.22 Diagnostic Model for HF

The construction of the nomogram was achieved based on the two biomarkers through the application of the “rms” package in R software. The assessment of diagnostic performance for HF entailed the generation of a receiver operation characteristic (ROC) curve to examine the nomogram. In addition, calibration curves and a decision curve analysis (DCA) enabled the evaluation of the predictive efficiency of the nomogram in HF.

### 2.23 Statistical Analysis

Statistical analyses were conducted utilizing GraphPad Prism 9.5 software. Measurement data were expressed as mean ± standard deviation, while categorical data were presented as counts (percentages). To assess the significance between groups, student's t‐test (two‐tailed) for comparisons between two groups, and one‐way analysis of variance (ANOVA) followed by Least Significant Difference (LSD) test (for equal variances) or Dunnett's test (for unequal variances) for comparisons involving three or more groups. Experiments with two categorical independent variables and one dependent variable were analyzed using two‐way ANOVA, followed by Bonferroni's multiple comparison tests. The numbers of sample sizes for each experiment were indicated in the figure legend. P‐values < 0.05 were considered significant, and significance for all figures was determined as ^*^ *p* < 0.05, ^**^ *p* < 0.01, ^***^ *p* < 0.001, and ^****^ *p* < 0.0001.

### 2.24 Ethics Approval Statement

All experiments involving humans were carried out in accordance with the Declaration of Helsinki. All procedures were performed in compliance with relevant laws and institutional guidelines. Ethics approval for the use of human plasma samples was provided by the Ethics Committee of Shaoxing Second Hospital (Ethics batch number: 2024052). Written informed consent was obtained from all patients before sample collection. And all animal experiments were approved by the Experimental Animal Ethics Committee of Ningbo University (batch number: 12654).

## 3 Results

### 3.1 CMS Induces Cardiomyocyte Hypertrophy in Rats

**Figure 1:**
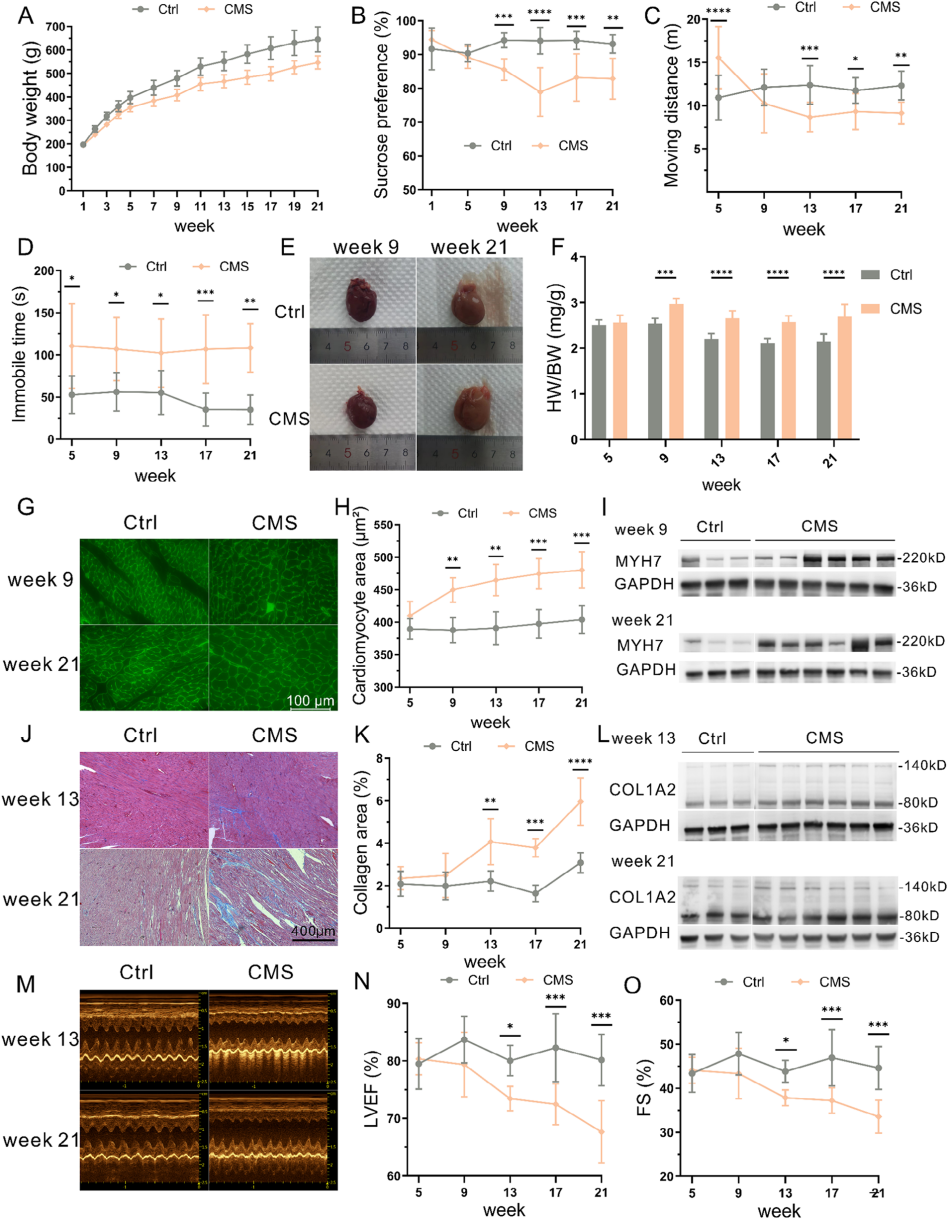
CMS induces cardiomyocyte hypertrophy in rats. A) Weight changes of control and CMS rats at 21 weeks (n=8‐40). B) Line graph showing sucrose intake ratio in control and CMS rats in sucrose preference test (n=5‐10). C) Line graph showing distance moved in the open field test for control and CMS rats in 5 min (n=6‐10). D) Line graph showing immobility time in the forced swimming test for control and CMS rats in 4 min (n=5‐8). E) Gross images of the heart at week 9 and 21 in control and CMS rats. F) Line graph showing heart weight‐to‐body weight ratio in 5 batches of control and CMS rats (n=5‐10). G) Representative images of WGA staining in the heart at weeks 9 and 21 in control and CMS rats. H) Quantitative analysis of WGA‐labeled cardiomyocyte area in 5 batches of control and CMS rats (n=4‐5). I) Expression of MYH7 protein in control and CMS rats at weeks 13 and 21. J) Staining for Masson's trichrome in control and CMS rats at weeks 13 and 21. K) Proportion of collagen fibers in 5 batches of control and CMS rats (n=4‐6). L) Expression of COL1A2 protein in control and CMS rats at weeks 13 and 21. M) Representative echocardiographic images of weeks 13 and 21 in control and CMS rats. N) Trend of LVEF changes in 5 batches of control and CMS rats (n=4‐10). O) Trend of FS changes in 5 batches of control and CMS rats (n=4‐10). CMS, chronic mild stress; WGA, wheat germ agglutinin; MYH7, myosin heavy chain 7; COL1A2, collagen type I alpha 2 chain; LVEF, left ventricular ejection fraction; FS, fractional shortening.

We conducted five batches of CMS modeling lasting 21 weeks to observe the pathological processes in the heart due to chronic psychological stress. First, we assessed the behavioral changes in the CMS group of rats to confirm the level of stress on the mental aspect. Rats in the CMS group showed a significant decrease in body weight compared to the control group after the onset of stress (**Figure** **1**A); the sucrose preference test showed a significant decrease in sucrose intake in the CMS group at week 9 and continued until the end of the experiment (Figure 1B), indicating anhedonia in the CMS rats; the open field test showed that the distance covered by the CMS group rats in 5 min was higher in the early stage than the control group, but lower in the later stage (Figure 1C), suggesting anxiety/depression in the CMS group; the forced swimming test showed a significantly longer immobility time in the CMS group rats within 6 min compared to the control group (Figure 1D), indicating a sense of hopelessness in the CMS rats.

**Figure 2:**
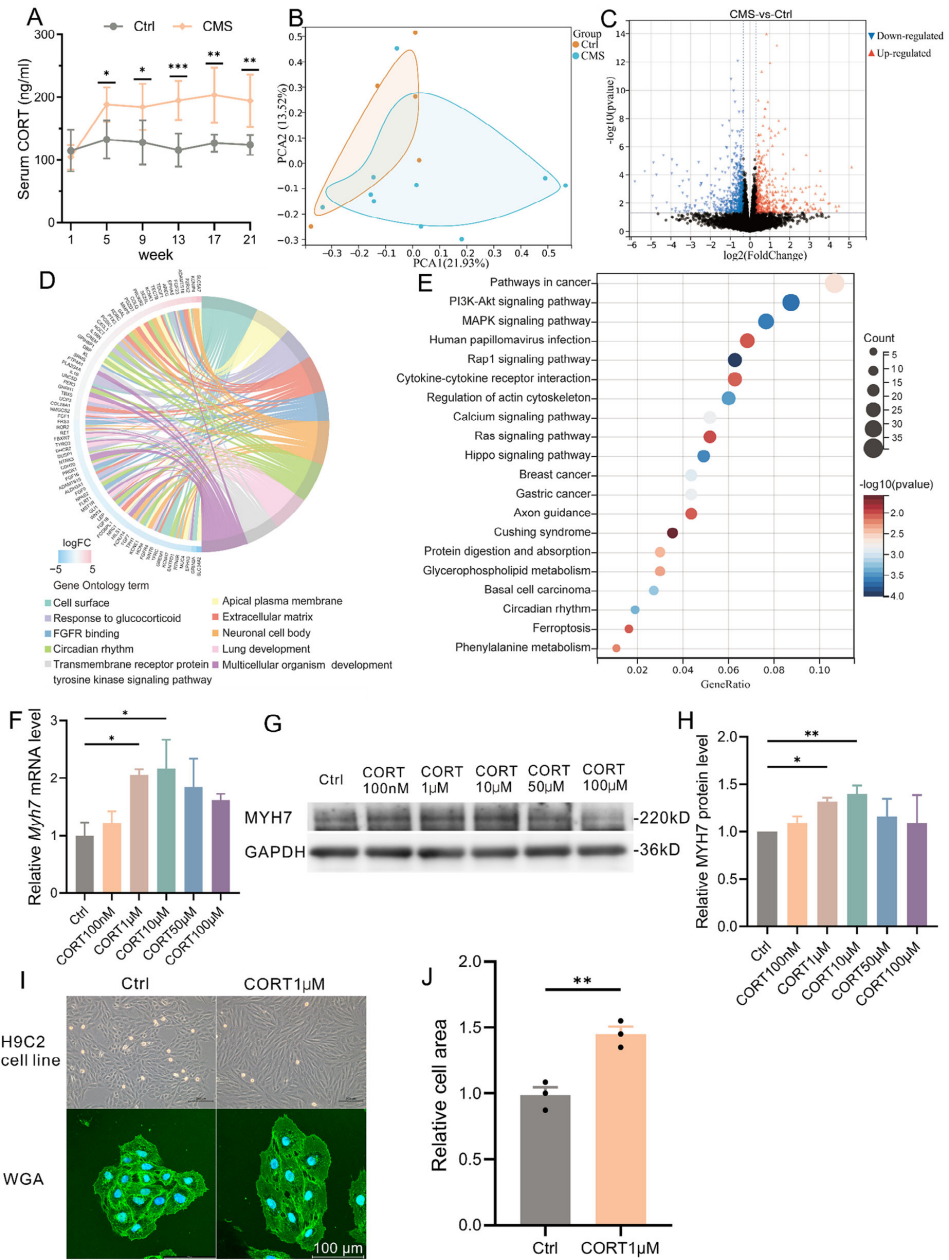
CORT may play an important role in CMS‐induced cardiomyocyte hypertrophy. A) Trend of serum CORT levels in 5 batches of control and CMS rats (n=4–8). B) PCA plot showing gene expression similarity between the control and CMS groups. C) Volcano plot displaying differential gene expression between control and CMS groups. D) Venn diagram showing the top 10 enriched GO terms from differential gene analysis. E) Bubble plot showing the top 20 KEGG‐enriched pathways from differential gene analysis. F) Relative mRNA expression of *Myh7* (n=3) in H9C2 cells stimulated with different concentrations of CORT for 48 h. G, H) Representative Western blot image (G) and quantification analysis (H) of MYH7 protein expression (n=3) in H9C2 cells stimulated with different concentrations of CORT for 48 h. I) Representative images of cell morphology (top) and WGA staining (bottom) in H9C2 cells stimulated with 1 µm CORT for 48 h. J) Quantitative analysis of cell surface area in WGA staining between control and CORT groups (n = 3). CMS, chronic mild stress; PCA, principal component analysis; GO, Gene Ontology; KEGG, Kyoto Encyclopedia of Genes and Genomes; MYH7, myosin heavy chain 7; CORT, corticosterone; WGA, wheat germ agglutinin.

Next, we evaluated the pathological changes in the heart from various aspects. Figure 1E,F show that the heart weight‐to‐body weight ratio in the CMS group rats started to increase significantly from week 9 compared to the control group. WGA staining revealed a significant increase in the surface area of cardiac muscle cells in the CMS group at 9 weeks and continued until the end of the experiment (Figure 1G,H). WB results showed a significant upregulation of the hypertrophic marker MYH7 in the heart tissue of the CMS group at week 9 and continued until the end of the experiment (Figure 1I; Figure S1A–J, Supporting Information). Masson's staining revealed a significant increase in fibrous tissue in the cardiac muscle tissue of the CMS group at week 13 and continued until the end of the experiment (Figure 1J,K). WB results showed a significant upregulation of the fibrotic marker COL1A2 in the heart tissue of the CMS group at week 13 and continued until the end of the experiment (Figure 1L; Figure S2A–J, Supporting Information). Echocardiography results showed a progressive decrease in LVEF and FS in the hearts of the CMS group rats, with a significant difference from week 13 (Figure 1M–O). However, there were no significant differences in diastolic interventricular septum thickness and left ventricular posterior wall thickness between the two groups throughout the study (Figure S3A,B, Supporting Information). To further clarify the changes in ventricular thickness, we performed HE staining of heart sections (Figure S3C–E, Supporting Information), which showed no statistically significant difference in ventricular wall thickness, although there was a trend of thickening in IVSd and LVPWd in the CMS group. We also observed changes in rat blood pressure and serum NTproBNP concentration. Blood pressure in the CMS group rats showed no significant difference compared to the control group in the first 4 batches, only to show a statistically significant increase in week 21 (Figure S4A, Supporting Information), while serum NTproBNP showed no significant difference between the two groups (Figure S4B, Supporting Information). Additionally, we measured the circulating concentrations of five stress‐related hormones, including CORT, adrenaline, serotonin, angiotensin II, and aldosterone. The results showed that CORT was consistently upregulated in CMS rats throughout the study (**Figure** **2**A), adrenaline significantly increased in the early stress period (week 5) and gradually decreased to normal levels, indicating a strong adaptability of the sympathetic nervous system in chronic psychological stress (Figure S4C, Supporting Information). The circulating levels of serotonin showed no significant differences in the five batches, while angiotensin II and aldosterone showed a trend of increase in the later stages of the study, but without statistical significance (Figure S4D–F, Supporting Information).

These results suggest that cardiomyocyte hypertrophy is an early and significant pathological phenotype in CMS rats. Therefore, we attempted to study the mechanisms and interventions for cardiomyocyte hypertrophy in CMS rats.

### 3.2 CORT Plays an Important Role in CMS Induced Cardiomyocyte Hypertrophy

**Figure 3:**
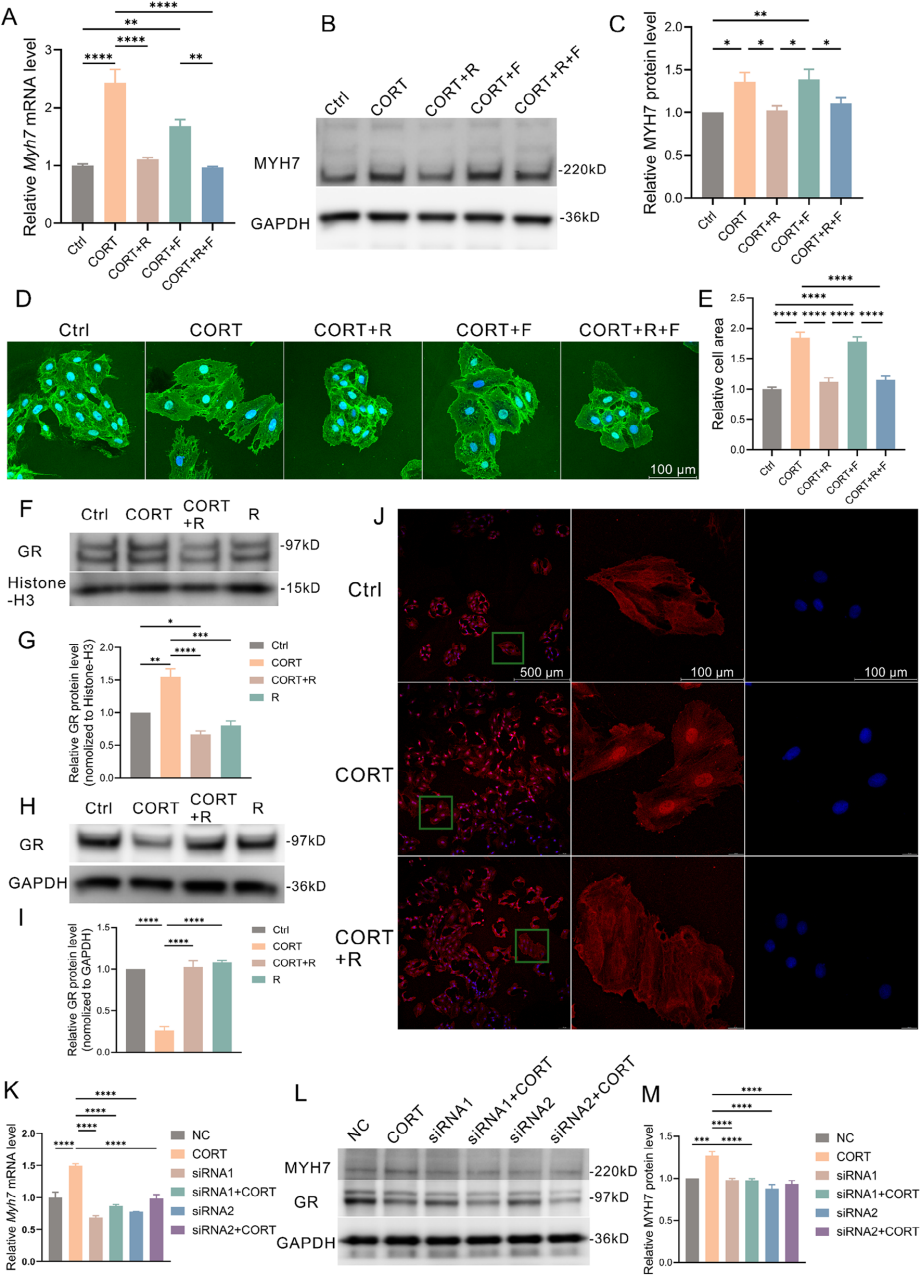
CORT induces H9C2 cell hypertrophy through GR. A) Relative mRNA expression levels of *Myh7* (n=3) in different groups of H9C2 cells. B, C) Representative Western blot image (B) and quantification analysis (C) of MYH7 protein expression (n=3) in different groups of H9C2 cells. D, E) Representative images of WGA staining (D) and quantification analysis (E) in different groups of H9C2 cells. F, G) Representative Western blot image (F) and quantification analysis (G) of nuclear GR protein expression (n=3) in different groups of H9C2 cells. H, I) Representative Western blot image (H) and quantification analysis (I) of cytoplasmic GR protein expression (n=3) in different groups of H9C2 cells. J) Representative images of GR immunofluorescence staining and DAPI staining in different groups of H9C2 cells. K) Relative mRNA expression levels of *Myh7* (n=3) in different groups of H9C2 cells. L, M) Representative Western blot image (L) and quantification analysis (M) of MYH7 protein expression (n=3) in different groups of H9C2 cells. GR, glucocorticoid receptor; MYH7, myosin heavy chain 7; CMS, chronic mild stress; PCA, principal component analysis; GO, Gene Ontology; KEGG, Kyoto Encyclopedia of Genes and Genomes; WGA, wheat germ agglutinin; CORT, corticosterone; R, relacorilant; F, finerenone.

In order to explore the pathological mechanism of cardiomyocyte hypertrophy in CMS rats, we extracted heart tissue for whole transcriptome sequencing. In the mRNA sequencing results, quality control analysis showed a high consistency in mRNA transcript abundance among the samples overall (Figure S5A,B, Supporting Information). 2D (Figure 2B) and 3D (Figure S5C, Supporting Information) PCA analysis revealed significant distribution differences between the CMS group and the control group. Differential gene analysis showed that in the hearts of CMS rats, 464 genes were significantly upregulated and 635 genes were significantly downregulated (Figure S5D, Supporting Information). The overall situation of differential gene expression and the TOP 30 are respectively shown in volcano plots (Figure 2C) and heat maps (Figure S5E, Supporting Information). GO enrichment analysis of differential genes (Figure 2D) showed that among the top 10 entries, the most significant biological process was “response to glucocorticoid”. GSEA also found that the “response to glucocorticoid” pathway was significantly activated in the CMS group (Figure S5F, Supporting Information). KEGG analysis (Figure 2E) revealed that differential genes were enriched in hypertrophy‐related signaling pathways such as “PI3K/AKT signaling pathway”, and a previous study has shown that the continuous activation of AKT is the main factor of volume‐loaded myocardial hypertrophy.^[^ ^14^ ^]^

In miRNA sequencing, the expression abundance of miRNAs showed high consistency (Figure S6A,B, Supporting Information), and PCA plots indicated minimal differences between groups (Figure S6C, Supporting Information). Differential expression analysis revealed significant differences in the expression levels of 15 miRNAs between groups (Figure S6D, Supporting Information). Prediction of target genes for these 15 differential miRNAs and functional enrichment analysis of the related target genes showed in KEGG secondary classification (Figure S7A, Supporting Information) that the top three organ systems enriched with target genes were “nervous system,” “endocrine system,” and “circulatory system”. KEGG pathway analysis further demonstrated (Figure S7B, Supporting Information) that the target genes of differential miRNAs were involved in various cardiomyopathy, such as “dilated cardiomyopathy” and “hypertrophic cardiomyopathy”. Additionally, KEGG analysis again highlighted pathological manifestations related to high glucocorticoid expression, such as “Cushing's syndrome” and “synthesis and secretion of cortisol”. In previous studies, it was found that glucocorticoids such as dexamethasone induce cardiomyocyte hypertrophy^[^ ^15^ ^]^; therefore, we believe that in CMS rats, sustained excessive secretion of CORT may be a promoting factor for cardiomyocyte hypertrophy.

To validate whether CORT has a pro‐hypertrophic effect on cardiomyocytes, experiments were conducted in the rat cardiomyocyte cell line H9C2. Treatment with CORT at concentrations ranging from 100nm to 100µm for 48 h, qPCR results showed (Figure 2F) that concentrations of 1–50µm CORT significantly increased the mRNA expression of the hypertrophic marker *Myh7* in H9C2 cells. Western blot results indicated (Figure 2G) that stimulation with 1 and 10µm CORT significantly increased the expression of MYH7 protein in H9C2 cells. Under the microscope (Figure 2I), it was evident that treatment with 1µm CORT induced significant morphological changes in H9C2 cells, as shown by WGA staining analysis (Figure 2J). 48 h of treatment with 1µm CORT resulted in a ≈40% increase in the cell surface area of H9C2 cells.

### 3.3 CORT Induces Hypertrophy Through Targeting GR in H9C2 Cells

CORT is a primary glucocorticoid secreted by the adrenal glands of rodents. It can exert effects by binding to both GR and mineralocorticoid receptors (MR), as well as unclear non‐genomic effects.^[^ ^16^ ^]^ Previously used GR antagonists and MR antagonists mainly include mifepristone and spironolactone, where mifepristone acts as an antagonist to GR and progesterone receptors, and spironolactone acts as an antagonist to MR and androgen receptors, both of which have strong side effects. Therefore, in this study, intervention was done using the novel selective GR antagonist, relacorilant (abbreviated to “R” in group naming), and the selective MR antagonist, finerenone (abbreviated to “F” in group naming). In vitro experiments in which drug interventions were performed, the following groups were included: control group (drug solvent treatment), CORT group (CORT 1µm), CORT+R group (CORT 1µm and relacorilant 1µm), CORT+F group (CORT 1µm and finerenone 10µm), CORT+R+F group (CORT 1µm, relacorilant 1µm, and finerenone 10µm), and R group (relacorilant 1µm). qPCR and WB results (**Figure** **3**A–C) showed that relacorilant significantly reduced the mRNA and protein expression of MYH7 induced by CORT, while finerenone could not effectively reverse the hypertrophic effect of CORT. Under the microscope (Figure S8A, Supporting Information), it was observed that relacorilant could significantly inhibit the morphological changes induced by CORT in H9C2 cells. The results of WGA staining (Figure 3D,E) suggested that relacorilant could antagonize the increase of H9C2 cell surface area induced by CORT, while finerenone had little effect. Since the activation of GR requires translocation into the nucleus to exert transcription factor effects, this process is termed “nuclear translocation.” Nuclear and cytoplasmic proteins were extracted for detection, and the results showed that after the addition of CORT, GR significantly increased in the cell nucleus (Figure 3F,G), significantly decreased in the cytoplasm (Figure 3H,I), and slightly decreased in total protein (Figure S8B,C, Supporting Information). This suggests that CORT promotes nuclear translocation and depletion of GR, while relacorilant may block this process. Cell immunofluorescence results (Figure 3J) showed that after the addition of CORT, GR concentrated internally in the cell nucleus, and relacorilant could effectively reverse the nuclear translocation caused by CORT. Additionally, two siRNAs with strong knockdown effects on GR were screened by qPCR (Figure S9A, Supporting Information). Quantification of cardiomyocyte cross‐sectional area by WGA staining (Figure S9B,C, Supporting Information) indicates that GR knockdown significantly alleviates CORT‐induced hypertrophy. qPCR and WB results (Figure 3K–M) showed that knockdown of GR significantly alleviated the upregulation of MYH7 expression induced by CORT.

Transcriptional abundance (Figure S10A, Supporting Information), PCA analysis (Figure S10B, Supporting Information), and cluster analysis (Figure S10C, Supporting Information) by H9C2 cells mRNA sequencing demonstrated intra‐group sample consistency and inter‐group variability, suggesting reproducibility of the intervention. Differential expression analysis (Figure S10D, Supporting Information) showed that compared to the control group, there were 455 upregulated genes and 187 downregulated genes in the CORT group; and compared to the CORT group, there were 182 upregulated genes and 431 downregulated genes in the CORT+R group, with the overall distribution of genes shown in volcano plots (Figure S10E,F, Supporting Information). GO analysis of the CORT versus control group (Figure S11A, Supporting Information) indicated that the enriched main BP items for differential genes included “dexamethasone cell response”, “integrin‐mediated signaling pathway”, and “extracellular matrix remodeling”, while CC items mainly included “extracellular matrix” and “laminin5 complex”, suggesting a strong effect of CORT intervention on extracellular matrix remodeling in cardiomyocytes. KEGG analysis (Figure S11B, Supporting Information) showed that differential genes were highly enriched in heart diseases such as “hypertrophic cardiomyopathy”, “dilated cardiomyopathy”, as well as pathways like “ECM‐receptor interaction” and “PI3K‐AKT signaling pathway”. Enrichment analysis of the CORT+R group compared to the CORT group showed results similar to the CORT versus control group findings (Figure S11C,D, Supporting Information). GSEA further demonstrated that pathways related to cardiomyocyte hypertrophy, such as “hypertrophic cardiomyopathy”, “dilated cardiomyopathy”, and “PI3K‐AKT signaling pathway”, were activated in the CORT group (Figure S11E,F, Supporting Information).

### 3.4 GR Promotes the Expression of Its Direct Target Gene (*Lama5*)

**Figure 4:**
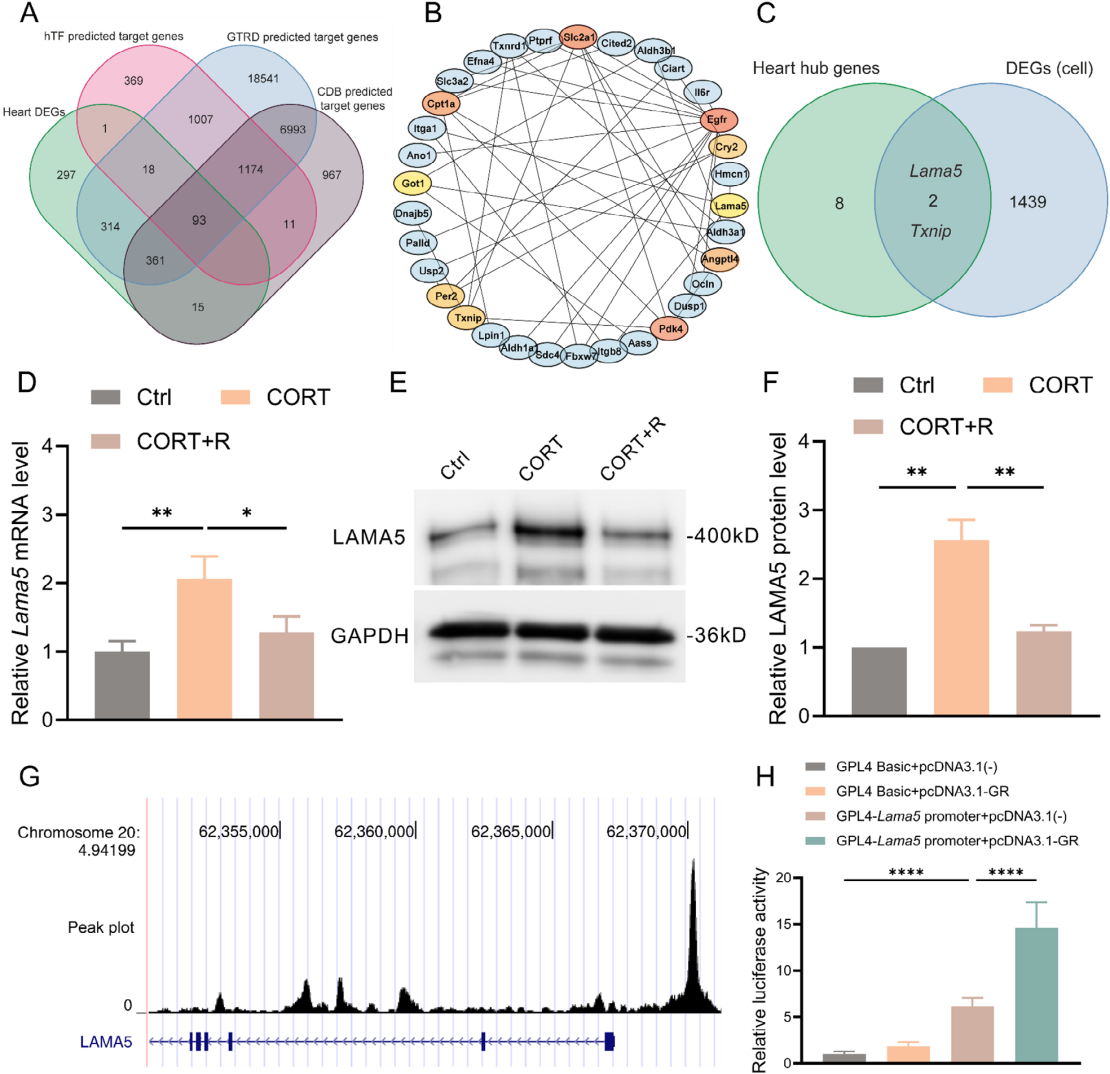
*Lama5* is a direct target gene of GR. A) Venn diagram showing 93 intersection genes between differentially expressed genes in rat heart tissue and GR target genes predicted by hTF, GTRD, and CDB databases. B) PPI analysis of the intersection genes, showing the common genes (marked in blue) and the top 10 hub genes (not marked in blue). C) Venn diagram showing the intersection of genes (*Lama5* and *Txnip*) between hub genes and differentially expressed genes in H9C2 cells. D–F) Relative mRNA expression levels (D), representative Western blot image (E), and quantification analysis (F) of LAMA5 protein in H9C2 cells (n=3). G) ChIP‐Seq analysis in muscle cells treated with CORT from the CDB database, showing the binding regions of GR with *Lama5* DNA. H) Dual‐luciferase reporter gene assay showing the relative luciferase intensity among different treatment groups (n=3). LAMA5, laminin subunit alpha 5; GR, glucocorticoid receptor; PPI, protein‐protein interaction; TXNIP, thioredoxin interacting protein; CORT, corticosterone; R, relacorilant; ChIP‐Seq, chromatin immunoprecipitation‐sequencing.

As a transcription factor, GR participates in the transcriptional activation or inhibition of multiple downstream genes, and there is currently no clear association between known GR target genes and cardiomyocyte hypertrophy. Therefore, we attempted to identify new potential target genes involved in GR‐induced cardiomyocyte hypertrophy. We predicted GR target genes using three transcription factor databases, including database of human transcription factor targets (hTFtarget)^[^ ^17^ ^]^, gene transcription regulation database (GTRD)^[^ ^18^ ^]^ and cistrome data browser (CDB)^[^ ^19^ ^]^, intersecting them with differentially expressed genes from rat heart tissue transcriptome, resulting in 93 differentially expressed potential GR target genes (**Figure** **4**A). Analysis of protein‐protein interactions (PPI) was performed on the 93 potential target genes, and subsequently, utilizing the cytohuba algorithm to obtain the top 10 hub genes (Figure 4B). By intersecting the 10 hub genes obtained from the animal sequencing results and the GR predicted target gene set analysis with the differential gene set of cell sequencing, we identified the top 2 genes showing the most significant fold change in expression at the cellular level (*Lama5* and *Txnip*). LAMA5 is one of the non‐collagen components of the extracellular matrix^[^ ^20^ ^]^, and until recent years, there has been no related research in the field of heart disease. In 2023, a study on heart‐like organs discovered that LAMA5 is an important factor for the development and maturation of cardiac muscle cells, and its deficiency can lead to developmental abnormalities in fetal hearts.^[^ ^21^ ^]^ Therefore, we are highly interested in investigating whether *Lama5* is a direct target gene of GR and its role in cardiomyocytes. The results of qPCR and WB (Figure 4D–F) revealed that CORT intervention significantly increased the levels of *Lama5* mRNA and protein expression, and this change could be reversed by using the GR antagonist, relacorilant. Furthermore, results from ChIP‐Seq indicated a high binding rate between GR and the gene sequence where *Lama5* is located (Figure 4G). Results from a dual luciferase reporter gene assay (Figure 4H) demonstrated that the artificially constructed promoter sequence of Lama5 has the ability to initiate downstream luciferase expression, and increasing GR levels can enhance the transcriptional activity of the *Lama5* promoter, significantly increasing luciferase expression.

### 3.5 CORT‐GR Partially Induces Cardiomyocyte Hypertrophy Through LAMA5

**Figure 5:**
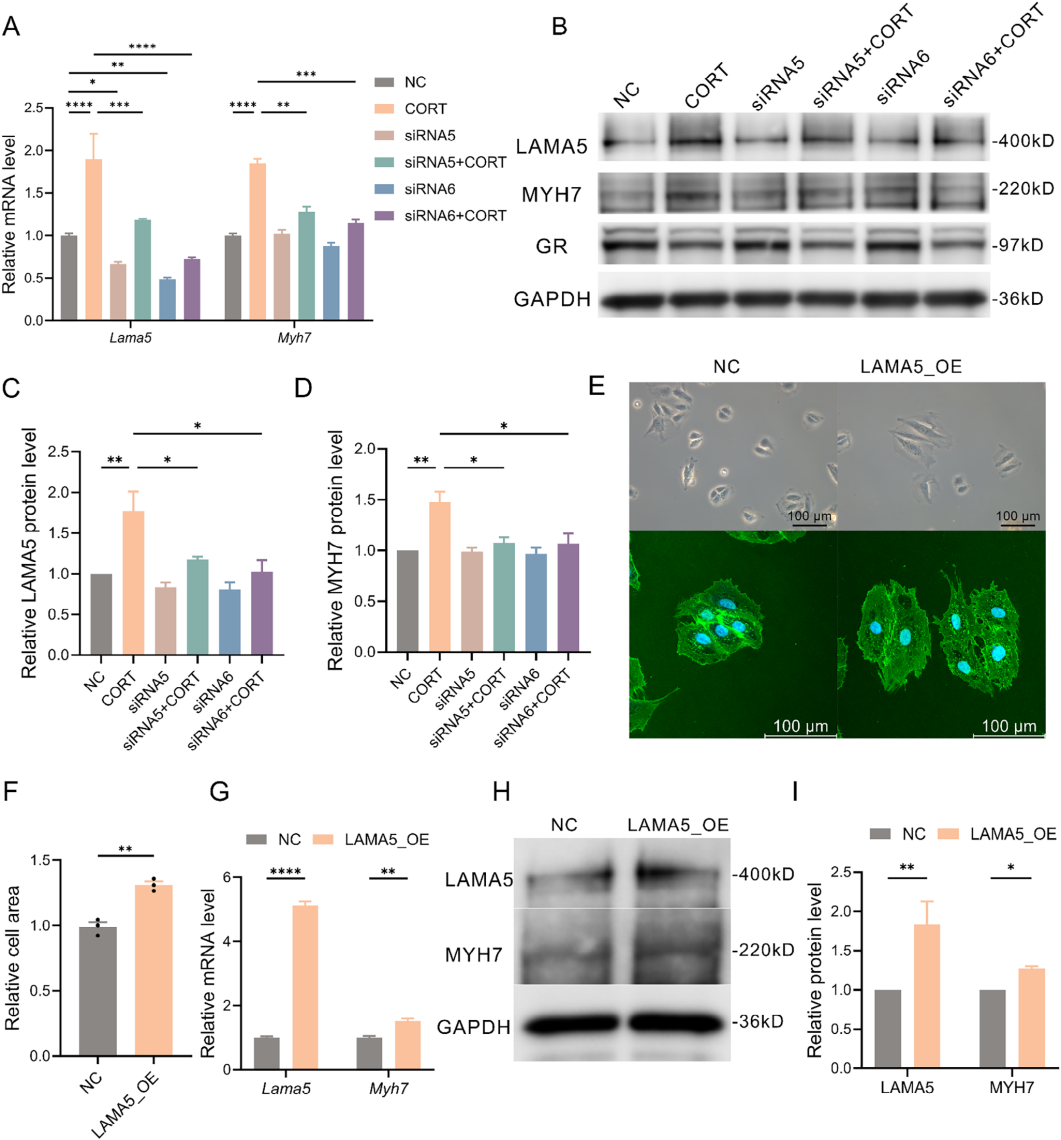
CORT/GR can induce cardiomyocyte hypertrophy through LAMA5. A) Relative mRNA expression levels of *Lama5* and *Myh7* in different treatment groups of H9C2 cells (n=3‐4). B) Representative Western blot images of LAMA5, MYH7, and GR in different treatment groups of H9C2 cells. C, D) Quantitative analysis of LAMA5 (C) and MYH7 (D) protein expression in different treatment groups of H9C2 cells (n=3). E) Representative cell morphology (top) and WGA staining images (bottom) of LAMA5‐overexpressing stable cells and NC group H9C2 cells. F) Quantitative analysis of WGA staining in *Lama5*‐overexpressing stable cells and NC group H9C2 cells (n=3). G–I) Relative mRNA expression levels of *Lama5* and *Myh7* (G), representative protein Western blot images (H), and quantitative analysis (I) in *Lama5*‐overexpressing stable cells and NC group H9C2 cells (n=3‐4). CORT, corticosterone; GR, glucocorticoid receptor; LAMA5, laminin subunit alpha 5; MYH7, myosin heavy chain 7; WGA, wheat germ agglutinin; NC, negative control.

Four siRNAs targeting rat *Lama5* were designed, with qPCR results (Figure S12A, Supporting Information) indicating that siRNA5 and siRNA6 achieved higher knockdown efficiency. Quantification of cardiomyocyte cross‐sectional area by WGA staining (Figure S12B,C, Supporting Information) indicates that *Lama5* knockdown significantly alleviates CORT‐induced hypertrophy. qPCR results showed (**Figure** **5**A) that siRNA intervention for *Lama5* significantly reduced the mRNA expression levels of *Lama5* and inhibited the upregulation of *Myh7* induced by CORT. At the protein level, the addition of the two siRNAs only slightly decreased the baseline level of LAMA5, but significantly reduced the further upregulation of LAMA5 induced by CORT and inhibited the upregulation of MYH7 protein expression induced by CORT (Figure 5B–D). Furthermore, we established a cell line with overexpression of LAMA5, where, under the microscope, some cells exhibited noticeable morphological signs of cellular hypertrophy (Figure 5E), and WGA staining revealed an average increase in cell surface area of 1.3 times compared to the control group (Figure 5F). qPCR results showed (Figure 5G) that in the stable *Lama5* transfectants, the mRNA level of *Lama5* was four times higher than that of the control group, and the expression level of *Myh7* increased by ≈80%. WB results indicated (Figure 5H,I) that in the stable *Lama5* transfectants, the protein level of LAMA5 increased by 80%, and the MYH7 protein expression level increased by around 20%.

### 3.6 Inhibition of LAMA5 Rescues Cardiomyocyte Hypertrophy In Vivo

**Figure 6:**
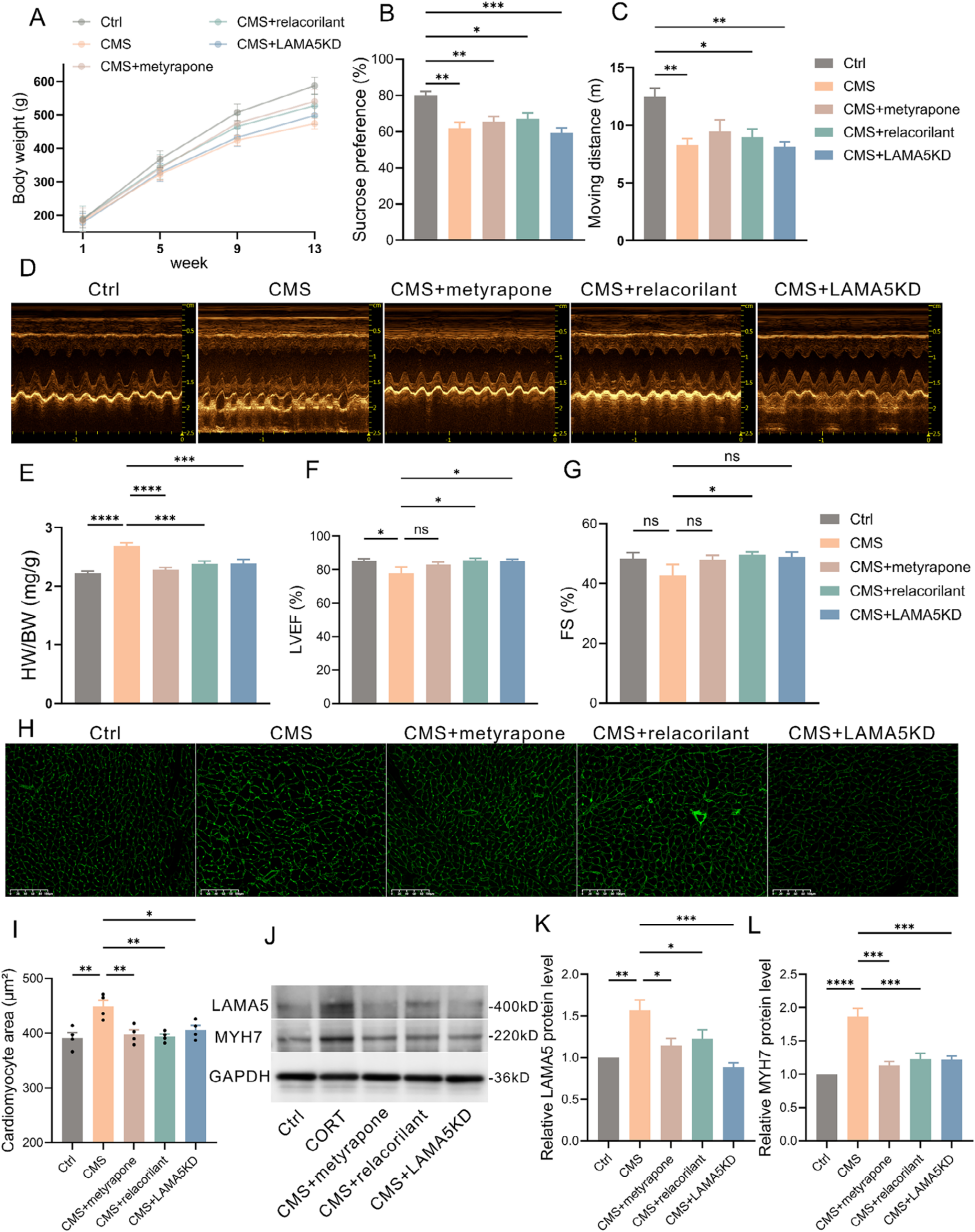
Chronic psychological stress induces cardiomyocyte hypertrophy and cardiac dysfunction through the CORT‐GR‐LAMA5 axis. A) Trend of body weight changes in each group of rats (n=7‐8). B) Sucrose preference ratio at the end of the experiment in each group of rats (n=4). C) 5‐min distance moved in the open field test at the end of the experiment in each group of rats (n=5). D) Representative images of echocardiography at the end of the experiment in each group of rats. E–G) HW/BW (E), LVEF (F), and FS (G) at the end of the experiment in each group of rats (n=4‐8). H, I) Representative images (H) and quantitative analysis (I) of WGA staining in cardiac myocytes of each group of rats (n=4). J) Representative Western blot images of LAMA5 and MYH7 in heart tissue of each group of rats. K, L) Quantitative analysis of LAMA5 (K) and MYH7 (L) protein expression in heart tissue of each group of rats (n=3). GR, glucocorticoid receptor; LAMA5, laminin subunit alpha 5; MYH7, myosin heavy chain 7; HW/BW, heart weight/body weight; LVEF, left ventricular ejection fraction; FS, fractional shortening; WGA, wheat germ agglutinin.

To further verify whether the CORT‐GR‐LAMA5 axis could mediate cardiomyocyte hypertrophy induced by CMS, we used metyrapone to inhibit CORT synthesis and relacorilant to antagonize the GR receptor in the CMS animal model. Additionally, we constructed an AAV carrying the cTnT gene to achieve targeted knockdown of *Lama5* gene expression in rats' hearts. qPCR and WB results from rat hearts indicated (Figure S13A–C, Supporting Information) that the total mRNA level of *Lama5* in the hearts infected with *Lama5* knockdown (LAMA5KD) adeno‐associated virus was ≈50% of that in the control group, and the protein level was around 75% of that in the control group. The experimental groups included a control group, CMS group, CMS+metyrapone group, CMS+relacorilant group, and CMS+LAMA5KD group. The results showed (**Figure** **6**A) that the body weights of rats in the four stress groups decreased to varying degrees, with the CMS group and CMS+LAMA5KD group showing the greatest decrease. The sucrose preference test results (Figure 6B) showed a decreased sucrose intake ratio in the four stress groups compared to the control group, with statistically significant differences. In the open field test (Figure 6C), the average distance moved by rats in the CMS/CMS+relacorilant/CMS+LAMA5KD groups over 5 min was significantly shorter than that of the control group, with statistically significant differences. The heart weight/body weight ratio was increased in the CMS group compared to the control group, and interventions with metyrapone/relacorilant and LAMA5KD mitigated this change (Figure 6E). Echocardiography results showed that the LVEF and FS of rats in the CMS group were significantly decreased compared to the control group, and metyrapone/relacorilant/LAMA5KD could alleviate the cardiac dysfunction caused by CMS (Figures 6D–G). WGA staining results showed that the surface area of cardiomyocytes in the hearts of rats in the CMS group was significantly increased compared to the control group, and metyrapone/relacorilant/LAMA5KD could reverse the cardiomyocyte enlargement induced by CMS (Figures 6H,I). WB results showed that the expression levels of LAMA5 and MYH7 were significantly increased in the hearts of rats in the CMS group, while metyrapone and relacorilant could inhibit the high expression of LAMA5 and MYH7 induced by CMS. In the CMS+LAMA5KD group, the expression of LAMA5 was decreased, and the protein level of MYH7 showed no significant difference compared to the control group (Figure 6J–L). Masson's trichrome staining revealed significant interstitial fibrosis in the hearts of rats in the CMS group compared to the control group, with similar fibrotic changes observed in the CMS+LAMA5KD group. Interestingly, the CMS+metyrapone and CMS+relacorilant groups could reverse the myocardial fibrosis induced by CMS (Figure S13D,E, Supporting Information).

### 3.7 Correlation of Serum CORT and LAMA5 with Cardiac Function in Wistar Rats

**Figure 7:**
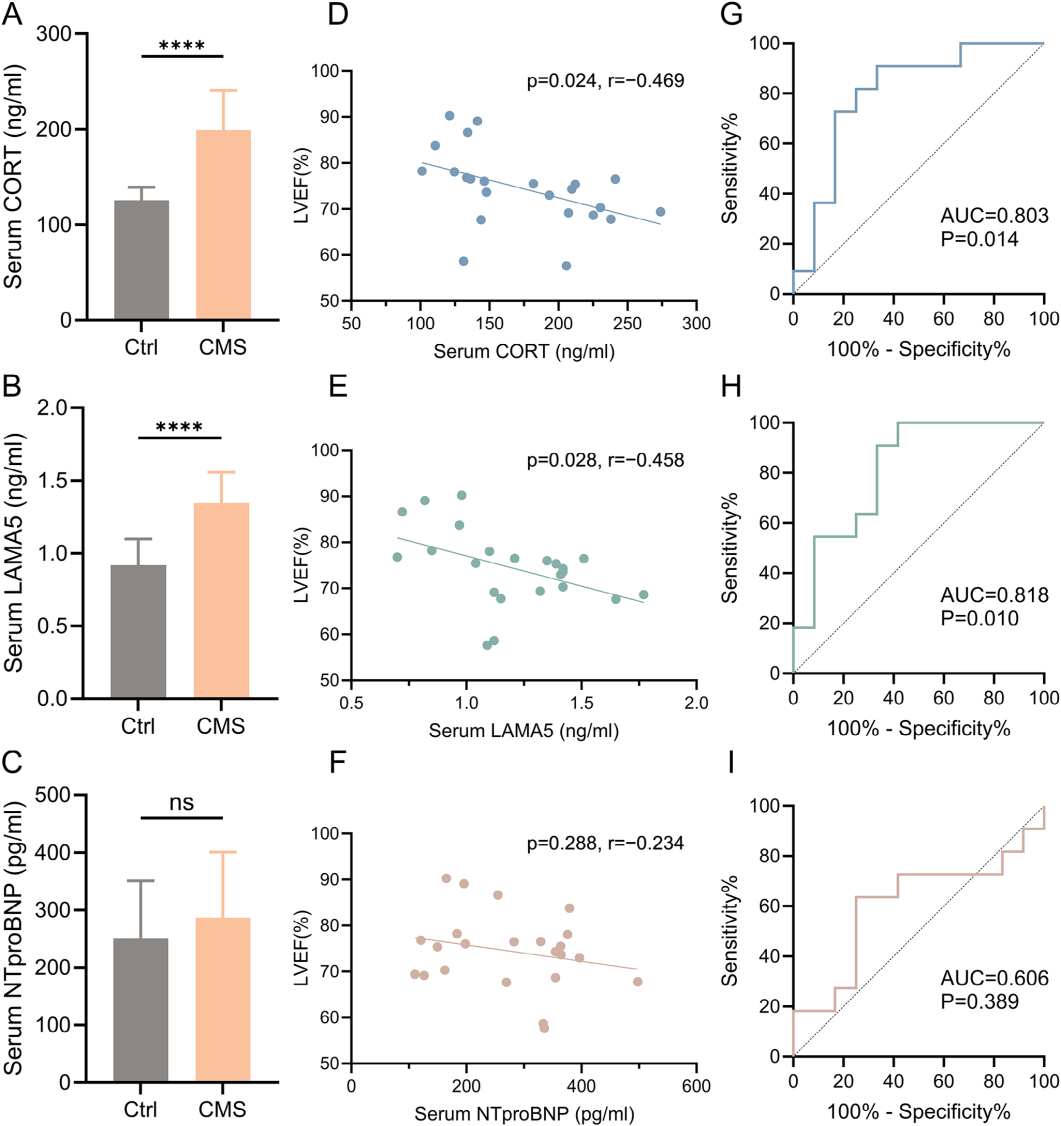
Correlation between serum CORT and LAMA5 levels with cardiac function in Wistar rats. A–C) Concentration analysis of serum CORT (A), LAMA5 (B), and NTproBNP (C) in control and CMS rats (n=8‐15). D–F) Scatter plots showing the correlation between serum CORT (D), LAMA5 (E), and NTproBNP (F) levels with LVEF in rats (n=23). G–I) ROC analysis of serum CORT (G), LAMA5 (H), and NTproBNP (I) levels for the diagnosis of decreased LVEF in rats. CORT, corticosterone; LAMA5, laminin subunit alpha 5; NTproBNP, N‐terminal pro‐B‐type natriuretic peptide; CMS, chronic mild stress; LVEF, left ventricular ejection fraction; ROC, receiver operation characteristic.

Given the hypertrophic effect of CORT and LAMA5 in CMS rats, we measured the serum concentrations of CORT and LAMA5 in the fourth and fifth batches of modeled rats to assess their biomarker value. As a comparison, we also measured the serum concentration of NTproBNP. The results showed that the serum levels of CORT and LAMA5 in the CMS group rats were significantly elevated compared to the control group, while the difference in NTproBNP levels was not statistically significant (**Figure** **7**A–C). The serum levels of CORT and LAMA5 were significantly negatively correlated with LVEF, with correlation coefficients of −0.469 and −0.458, respectively, while NTproBNP showed no significant correlation with LVEF (Figure 7D–F). ROC analysis revealed that the serum levels of CORT and LAMA5 had AUC values of 0.803 and 0.818, respectively, for diagnosing decreased LVEF, whereas NTproBNP had an area under the curve (AUC) of 0.606 for diagnosing decreased LVEF (Figure 7G–I).

### 3.8 LAMA5 is a Novel Diagnostic Marker for Myocardial Hypertrophy in Patients with Depression

**Figure 8:**
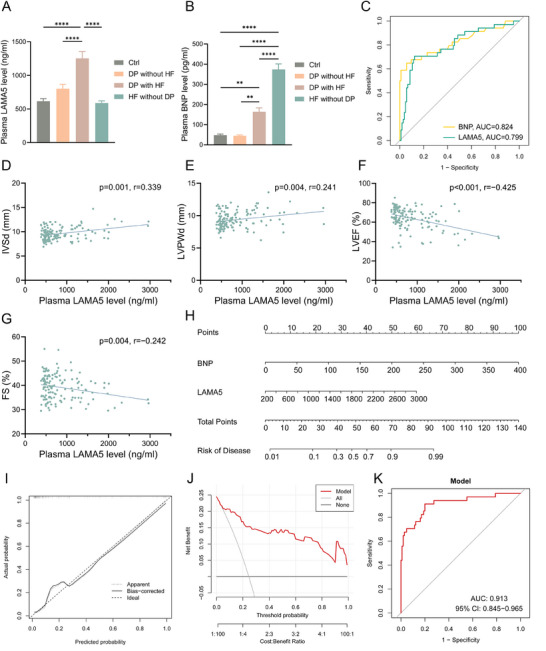
Correlation and diagnostic value of plasma LAMA5 levels with cardiac function in patients with DP. A, B) Plasma levels of LAMA5 (A) and BNP (B) in patients in each group (n=34‐62). C) ROC analysis of BNP and LAMA5 for diagnosing DP with HF. D–G) Scatter plots showing the correlation between plasma LAMA5 levels in patients with IVSd (D), LVPWd (E), LVEF (F), and FS (G) (n=139). H) Forest plot of BNP and LAMA5 for diagnosing DP with HF. I–K) Calibration curve of diagnostic model (I), DCA analysis (J), and ROC analysis (K). LAMA5, laminin subunit alpha 5; DP, depression; BNP, B‐type natriuretic peptide; ROC, receiver operation characteristic; HF, heart failure; IVSd, diastolic interventricular septal; LVPWd, diastolic left ventricular posterior wall; LVEF, left ventricle ejection fraction; FS, fractional shortening; DCA, decision curve analysis.

In order to explore the biomarker value of glucocorticoids and LAMA5 in chronic psychological stress‐induced changes in cardiac structure and function, we recorded clinical data from DP without HF, DP with HF, HF without DP, and healthy control groups, and collected plasma samples for the determination of cortisol and LAMA5 concentrations. The baseline data of each group are shown in Table 1, with no significant differences in non‐disease‐specific indicators such as age and gender. As shown in **Figure** **8**A, compared to the other groups, LAMA5 was significantly elevated in the DP with HF group, moderately elevated in the DP without HF group, while the levels of LAMA5 in the HF without DP group did not show significant differences compared to the control group. Cortisol concentration was higher in the DP with HF group and the DP without HF group compared to the control group (Figure S14A, Supporting Information). BNP levels were slightly elevated in the DP with HF group and significantly elevated in the HF without DP group (Figure 8B). To further explore the diagnostic value of cortisol and LAMA5 for cardiac function in patients with depression, we conducted ROC and correlation analyses in patients of the control group, DP with HF group, and DP without HF group. The diagnostic ROC analysis for HF showed AUC values of 0.799 for LAMA5 and 0.646 for cortisol in diagnosing DP with HF (Figure S14B, Supporting Information), while the diagnostic value for BNP was high at 0.824 (Figure 8C). LAMA5 had a significant positive correlation with IVSd and LVPWd and a significant negative correlation with LVEF and FS (Figure 8D–G). Cortisol showed a significant negative correlation with LVEF, but no statistical significance in correlation with IVSd, LVPWd, and FS (Figure S14C–F, Supporting Information). To explore the diagnostic model for DP with HF, we included multiple factors such as age, gender, underlying diseases, lipid levels, and blood sugar levels for LASSO regression to select the main variables (Figure S14G, Supporting Information). The optimal diagnostic indicators were identified to be two, namely LAMA5 and BNP. The diagnostic nomogram is displayed in Figure 8H, calibration curves indicated relative reliability of the model (Figure 8I), DCA analysis showed positive clinical decision benefits for patients (Figure 8J), and the ROC analysis demonstrated a diagnostic AUC of 0.913 for the model (Figure 8K).

### 3.9 The CORT‐GR‐LAMA5 Axis may Induce Cardiomyocyte Hypertrophy Regulating the ITGB1‐PI3K‐AKT Pathway

**Figure 9:**
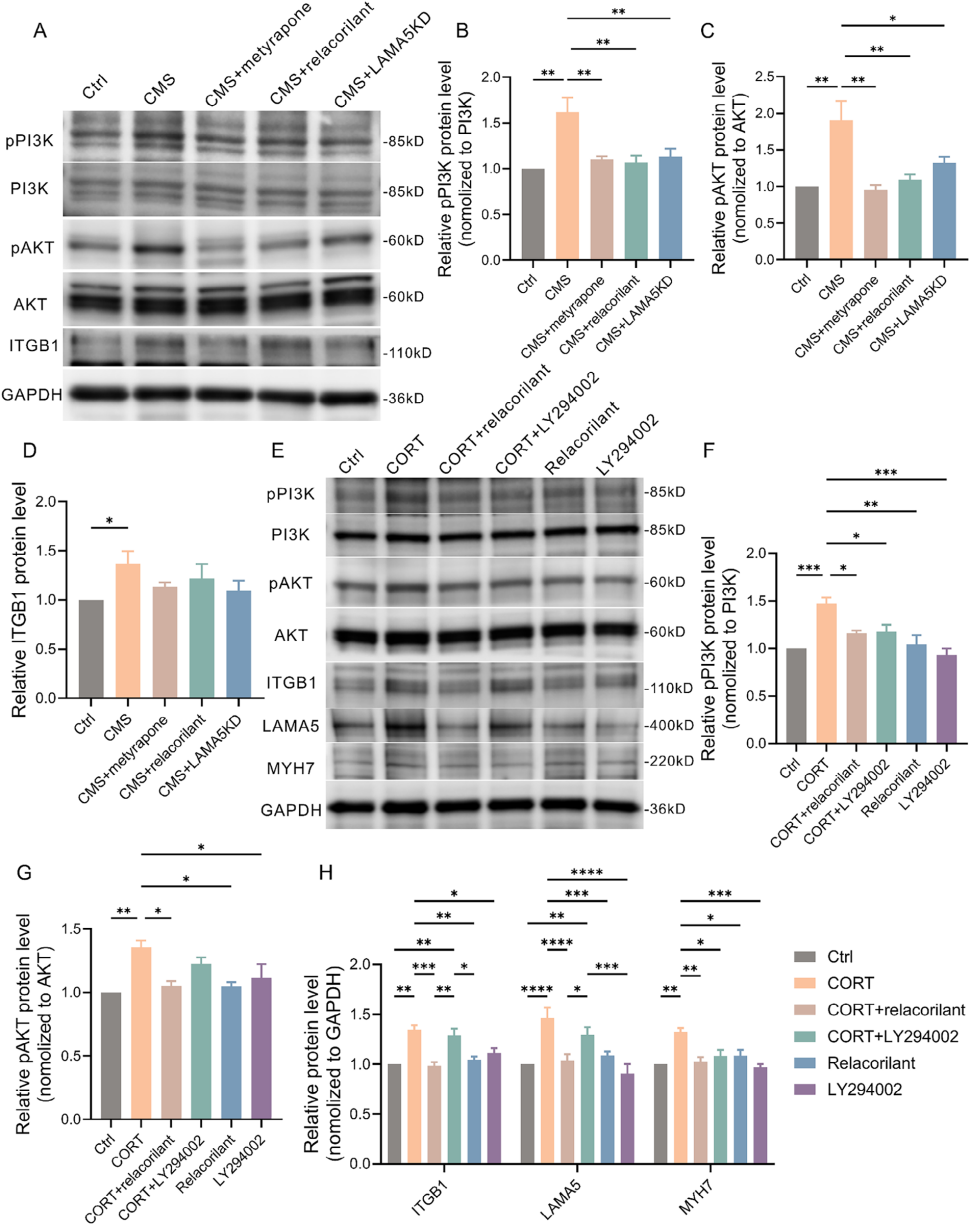
The CORT‐GR‐LAMA5 axis may induce cardiomyocyte hypertrophy through the ITGB1‐PI3K‐AKT pathway. A) Representative Western blot images of pPI3K, PI3K, pAKT, AKT, and ITGB1 in heart tissue of each group of rats. B–D) Relative protein expression levels of pPI3K (B), pAKT (C), and ITGB1 (D) in heart tissue of each group of rats (n=3). E) Representative Western blot images of PI3K/AKT/MYH7/LAMA5 in H9C2 cells of each group. F, G) Quantitative analysis of relative protein expression levels of pPI3K (F) and pAKT (G) in H9C2 cells of each group (n=3). H) Quantitative analysis of protein expression levels of ITGB1, LAMA5, and MYH7 in H9C2 cells of each group (n=3). CORT, corticosterone; GR, glucocorticoid receptor; LAMA5, laminin subunit alpha 5; ITGB1, integrin subunit beta 1; PI3K, phosphatidylinositol 3‐kinase; MYH7, myosin heavy chain 7.

LAMA5 is an important component of the extracellular matrix and can bind to various integrin receptors to initiate downstream pathways and exert physiological effects. In cell sequencing results, GO analysis enriched multiple integrin‐related pathways such as “integrin‐mediated signaling” and “integrin binding,” while KEGG analysis enriched pathways including “ECM‐receptor interaction” and “PI3K/AKT.” Numerous studies have indicated the significant role of the integrin‐PI3K‐AKT pathway in cardiomyocyte hypertrophy. Therefore, we hypothesize that CORT‐GR‐LAMA5‐induced cardiomyocyte hypertrophy may be related to the ITGB1‐PI3K‐AKT pathway. We examined the activation status of PI3K/AKT in the animal model and found that in the CMS group, there was an upregulation of ITGB1/GAPDH, pPI3K/PI3K, and pAKT/AKT compared to the control group, while metyrapone, relacorilant, and knockdown of LAMA5 partially inhibited the activation of the PI3K/AKT pathway (**Figure** **9**A–D). Furthermore, we treated the H9C2 cell line with relacorilan and the PI3K inhibitor LY294002, and the results are shown in Figure 9E–H. After CORT intervention, the expression of ITGB1, LAMA5, MYH7, pPI3K, and pAKT was upregulated in the cells. Relacorilant intervention inhibited the CORT‐induced activation of PI3K/AKT, reduced the expression of LAMA5 and ITGB1, and attenuated the excessive upregulation of MYH7. However, under CORT combined with LY294002 intervention, the expression levels of LAMA5 and ITGB1 remained elevated compared to the control group, while the activation of the PI3K/AKT pathway was inhibited, leading to a downregulation of MYH7, indicating that LY294002 could alleviate the CORT‐induced MYH7 overexpression.

## 4 Discussion

In today's fast‐paced world, chronic stressors are abundant. According to the GBD 2021 report, depressive disorders and anxiety disorders ranked 2nd and 6th, respectively, in terms of causes of years lived with disability (YLD) across all age groups, with the combined YLD from both (98.8 million) exceeding the top‐ranked low back pain (70.2 million).^[^ ^22^ ^]^ Therefore, further research is urgently needed in the field of anxiety/depression‐related physical and mental illnesses to address the increasing disease burden brought by a growing number of anxiety/depression patients. In the realm of cardiac structural diseases caused by chronic psychological stress, current research is relatively scarce, lacking comprehensive and continuous observations of disease progression. In this study, through five batches of chronic psychological stress lasting ≈5 months, we systematically observed the overall size/thickness, microscopic pathological manifestations, and functional changes of the hearts of CMS rats, and proposed for the first time that cardiomyocyte hypertrophy is the earliest cardiac pathological phenotype to appear throughout the entire CMS cycle, followed by mild interstitial fibrosis and contractile dysfunction. This is consistent with the common progression of various cardiomyopathies in clinical practice, where cardiomyocyte hypertrophy or myocardial fibrosis often appears earlier than the symptoms or signs of manifest cardiac dysfunction.^[^ ^23^ ^]^ Additionally, it is noteworthy that when severe depression symptoms such as anhedonia were observed in the 9th week, cardiomyocyte hypertrophy was also detected, suggesting that cardiomyocyte hypertrophy and severe depression might occur simultaneously. Therefore, it is important to consider whether cardiomyocyte hypertrophy is already present in patients with severe depression, and monitoring the cardiomyocyte hypertrophy is particularly crucial for the early identification and intervention of the cardiac pathological progression in patients with anxiety/ion.

However, the even more astonishing discovery is that in our rat model, cardiomyocyte hypertrophy appears earlier than the increase in blood pressure, indicating that the cardiomyocyte hypertrophy in CMS rats is not an adaptive change resulting from increased cardiac afterload. This phenomenon suggests the necessity to further explore the mechanisms of cardiomyocyte hypertrophy in CMS rats. Therefore, we conducted whole transcriptome sequencing of heart tissues from model and control rats to observe significant changes in molecular and biological processes. The enrichment analysis of differentially expressed mRNAs and miRNAs revealed the effects of glucocorticoids. Serum hormone testing in the rats from 5 batches revealed that NA increased in the early stages of stress but quickly recovered, indicating sympathetic adaptability in rats. Hormones such as 5HT/aldosterone/angiotensin II did not significantly increase in the early stages, while CORT increased significantly early in CMS and remained high until the end of the experiment. These results suggest that CORT may play an important role in cardiomyocyte hypertrophy induced by CMS. Studies have shown that the synthetic long‐acting glucocorticoid dexamethasone can induce hypertrophy in H9C2 cell lines and primary rat cardiomyocytes.^[^ ^15^ ^]^ Unfortunately, previous related studies have used RU486 as a GR antagonist in experiments. RU486 not only binds to GR but also to progesterone receptors (PR) to exert contraceptive effects, and its affinity for PR is 13 times that of GR.^[^ ^24^ ^]^ Therefore, the role of CORT‐GR axis in inducing cardiomyocyte hypertrophy in CMS rats remains unclear, and the clinical utility of RU486 is limited due to its contraceptive side effects. Thus, in this study, we used relacorilant, a novel selective GR antagonist, in experiments. Additionally, we added a metyrapone group and found that inhibiting CORT synthesis can alleviate CMS‐induced cardiomyocyte hypertrophy, confirming the significant role of the CORT‐GR axis in cardiomyocyte hypertrophy in CMS rats.

However, the specific mechanism by which CORT‐GR leads to cardiomyocyte hypertrophy is still unclear. Considering that GR is a transcription factor that is activated upon binding with CORT, playing a role in transcriptional activation or inhibition of downstream target genes by translocating into the nucleus.^[^ ^25^ ^]^ Therefore, we predicted GR target genes based on three databases and integrated tissue and cell sequencing data to ultimately identify 10 potential candidate target genes that play important roles in the heart. Among them, LAMA5 is a large (≈400 kD), evolutionarily conserved multi‐domain protein involved in forming the trimeric structure of laminin^[^ ^26^ ^]^, a major component of the extracellular matrix and basement membrane. Previous studies have shown that defects in LAMA5 lead to a variety of developmental abnormalities, including osteogenesis imperfecta, pulmonary hypoplasia, and nephrotic syndrome.^[^ ^27^ ^]^ Recent research has found that in cardiac‐like organs, LAMA5 is an important factor in promoting the maturation of cardiomyocytes, with a 20% decrease in myocardial contractile force following LAMA5 knockdown. Genetic knockout of LAMA5 is lethal during embryonic development, and mice carrying mutated LAMA5 genes (resulting in laminin polymerization defects) have a 20% reduction in the area of the left ventricular compact myocardium compared to wild‐type mice, suggesting the essential role of LAMA5 in the development and maturation of cardiomyocytes.^[^ ^21^ ^]^ Therefore, we have a strong interest in understanding the role of LAMA5 in the heart. Through transcription factor dual‐luciferase reporter gene experiments and ChIP‐Seq data, we confirmed that *Lama5* is a direct target gene of GR. Furthermore, through *Lama5* knockdown and overexpression cell experiments, we discovered the important role of the CORT‐GR‐LAMA5 axis in cardiomyocyte hypertrophy, and animal experiments have confirmed this finding. Notably, inhibiting CORT reduced myocardial fibrosis in CMS rats, but LAMA5 knockdown did not. This suggests CORT may activate cardiac fibroblasts independently of LAMA5. A recent study found myocardial fibrosis in Beagle dogs given 4mg kg^−1^ prednisolone (a synthetic glucocorticoid) for 84 days, which may explain reduced fibrosis in CMS rats treated with metyrapone or relacorilant, and further research is needed to understand the specific mechanism.^[^ ^28^ ^]^ In addition, an interesting finding is that reported genetic defects in LAMA5 lead to infantile nephrotic syndrome, and most of these patients are resistant to corticosteroids.^[^ ^29^ ^]^ Since LAMA5 is a direct target gene of corticosteroid‐GR, this seems to explain the reason why children with LAMA5 defects exhibit resistance to corticosteroids.

While we recognize the value of cardiomyocyte hypertrophy in identifying cardiac structural abnormalities caused by CMS, it is currently difficult to identify the occurrence of cardiomyocyte hypertrophy in clinical settings. Because myocardial cells are non‐regenerative cells, it is challenging to obtain cardiac tissue for histological observations in clinical practice, and non‐invasive tests such as electrocardiograms, echocardiograms, and cardiac magnetic resonance imaging mainly focus on overall structural changes in the heart, making it difficult to detect changes at the cellular level. As one of the main participants in CORT‐induced cardiomyocyte hypertrophy, the serological prognostic value of LAMA5 warrants further research. Therefore, we first analyzed the correlation and diagnostic value of LAMA5 with LVEF in rats at the animal level, compared to NTproBNP, and found that in rats with CMS‐induced cardiac dysfunction, LAMA5 had a higher diagnostic value for decreased LVEF than NTproBNP. Furthermore, we collected depressive patients with or without HF, as well as healthy individuals, and found that LAMA5 was significantly upregulated in depressed patients with HF, showing a significant negative correlation with LVEF. Using LASSO regression to screen LAMA5, BNP, cortisol, and various clinical indicators, we obtained a comprehensive model including LAMA5 and BNP. This model achieved a diagnostic ROC of 0.913 for depressive patients with HF, demonstrating high diagnostic value. It is notable that, in contrast to BNP, LAMA5 does not exhibit a significant increase in HF patients without depression, indicating that LAMA5 is not a universal biomarker for myocardial hypertrophy or HF. The common HF is typically induced by underlying cardiac disorders, such as hypertension, during which the activation of the sympathetic nervous system and the renin‐angiotensin‐aldosterone system is predominantly involved, which is less associated with glucocorticoids. Thus, we contend that LAMA5, as a direct target of glucocorticoids, might not participate in the cardiomyocyte hypertrophy process resulting from other diseases but rather serves as a specific biomarker for psychogenic hypertrophy. This requires further validation through clinical studies with larger sample sizes in the future.

In terms of treatment, we found that inhibiting CORT synthesis and GR antagonism can effectively inhibit cardiomyocyte hypertrophy and cardiac dysfunction caused by CMS. Among them, the new drug relacorilant is currently in multiple clinical trials for treating Cushing's syndrome, platinum‐resistant ovarian cancer, prostate cancer, and other diseases, showing promising therapeutic potential.^[^ ^30^, ^31^, ^32^ ^]^ Compared to RU486, relacorilant not only lacks progesterone antagonist‐related complications, but also exhibits weaker feedback elevation of glucocorticoids after treatment than RU486, and no similar prolongation of the QT interval as seen with RU486 has been observed, demonstrating good safety.^[^ ^33^ ^]^ Furthermore, epidemiological surveys and clinical studies over the last two decades have shown a significant increase in the incidence and mortality of cardiovascular diseases in patients with Cushing's syndrome or long‐term use of exogenous glucocorticoids. Even in patients without clinical evidence of cardiovascular disease, cardiac magnetic resonance imaging has detected left ventricular hypertrophy, fibrosis, diastolic and systolic dysfunction in some patients with Cushing's syndrome, once again highlighting the adverse cardiovascular effects of glucocorticoids.^[^ ^34^ ^]^ A recent study found that out of 56 patients with Cushing's syndrome, 28 exhibited signs of HF, and surgical treatment of the primary lesion led to the resolution of HF syndrome in 11 patients, suggesting that glucocorticoid‐induced myocardial changes may be reversible.^[^ ^35^ ^]^ However, there is limited research on drug interventions for Cushing's syndrome‐related heart diseases, and the therapeutic value of relacorilant for Cushing's syndrome and Cushing's syndrome‐related heart diseases is promising. Furthermore, LAMA5 is also one of the potential therapeutic targets for heart disease in patients with depression/Cushing's syndrome/exogenous glucocorticoid supplementation. Especially for patients receiving exogenous glucocorticoid supplementation, who require the therapeutic effects of GCs while also needing to prevent their adverse cardiac effects, LAMA5 may be a specific therapeutic target. However, there are currently no drugs targeting LAMA5, emphasizing the urgent need to develop small‐molecule drugs or miRNA formulations targeting LAMA5 for further research.

Furthermore, our study initially identified the ITGB1‐PI3K‐AKT signaling pathway as a potential downstream mediator of LAMA5‐induced cardiomyocyte hypertrophy. However, it is important to note that LAMA5, as a component of the extracellular matrix, interacts with various proteins such as collagen and fibronectin, which may also significantly contribute to cardiomyocyte hypertrophy. Despite this, there has been limited research on the interactions between LAMA5 and other extracellular matrix components. In recent years, the differentiation of induced pluripotent stem cells (iPSCs) into cardiomyocytes has been extensively explored, with extracellular matrix proteins emerging as crucial mediators in both iPSC differentiation and the maturation of iPSC‐derived cardiomyocytes (iPSC‐CMs).^[^ ^36^ ^]^ Burridge et al. investigated the capacity of several extracellular matrix proteins and peptides to facilitate the formation of human iPSC‐CM monolayers and promote iPSC‐CM differentiation, including recombinant vitronectin, a synthetic vitronectin peptide, and recombinant human laminin‐521 (which is synthesized from LAMA5). Among the substrates evaluated, only the laminin‐based matrix demonstrated the ability to support long‐term adhesion (exceeding 15 days) of the iPSC‐CM monolayer.^[^ ^37^ ^]^ Subsequently, Yap et al. conducted a comparative analysis of the efficacy of laminin‐521 alone versus a combination of laminin‐521 and laminin‐221 in promoting the differentiation of human embryonic stem cells (hESCs) into cardiomyocytes. The study revealed that hESC‐derived cardiomyocytes produced using the combination of laminin‐521 and laminin‐221 exhibited a significantly higher purity (≈80%–90% cardiomyocytes) compared to those produced with laminin‐521 alone (≈30%–50% cardiomyocytes).^[^ ^38^ ^]^ This finding underscores the substantial impact that the combination of extracellular matrix proteins can have on the differentiation and maturation of cardiomyocytes. Moreover, this approach may serve as an effective strategy for investigating the interaction mechanism of LAMA5 and other extracellular matrix proteins.

The limitations of this study mainly include the following aspects. First, despite conducting a 20‐week continuous CMS modeling, the decrease in LVEF in rats was not high enough to reach a level of overt HF, and NTproBNP did not significantly increase (although there was a gradual upward trend), therefore, the diagnostic value of LAMA5 for HF at the animal level is debatable. Second, it is difficult in clinical practice to find patients with clear heart dysfunction caused by depression. The patients we collected with depression comorbid with HF also had various underlying heart diseases, which introduced interference with the plasma levels of LAMA5. Thus, longer duration of CMS modeling to achieve significant heart dysfunction or new psychological stress models for stronger induction of cardiac changes, as well as high‐quality clinical studies with strict inclusion/exclusion criteria and long‐term follow‐up, are needed to explore the diagnostic value of LAMA5 in heart dysfunction caused by psychological stress. Moreover, considering the clinically intricate relationship between depression and HF, investigating the prognostic value of LAMA5 in HF patients comorbid with anxiety/depression may be a topic worth further exploration. In addition, due to the myocardial protective effect of estrogen, only male rats were used in the animal experiments in this study to avoid the interference of gender on the results, so the effect of gender on the cardiac pathological changes caused by CMS needs to be further studied and clarified. Lastly, the downstream mechanisms of LAMA5 in this study were only subject to relatively simple validation, and we plan to use iPSC‐derived cardiomyocytes and *Lama5* knockout mice to further explore the hypertrophic mechanism of LAMA5 in future studies.

## Conflict of Interest

The authors declare no conflict of interest.

## Author Contributions

C.Z. and Y.S. contributed equally to this work. C.Z., J.L., and J.Z. did conceptualization. C.Z., Y.S., and X.J. did methodology. C.Z., H.L., J.H., and Z.W. did investigation. J.L., J.Z., and C.H. did project administration. C.Z. prepared the original draft. J.L., H.Y., and Q.X. reviewed and did editing. J.L. and Y.S. did funding acquisition. All authors have read and approved the final manuscript.

## Supporting information



Supporting Information

## Data Availability

The data that support the findings of this study are available from the corresponding author upon reasonable request.
