# Supplementary material for: Chronic Psychological Stress Induces Cardiomyocyte Hypertrophy Through Corticosterone‐Glucocorticoid Receptor‐LAMA5 Axis
Source: Adv Sci (Weinh). 2025 Jul 11;12(36):e14659. doi: 10.1002/advs.202414659 (PMC12463095; doi:10.1002/advs.202414659)
Supplement: Supplementary file 1 — Supporting Information [file ADVS-12-e14659-s001.docx]

**Supplementary Tables and Figures**

Chronic Psychological Stress Induces Cardiomyocyte Hypertrophy Through Corticosterone-Glucocorticoid Receptor-LAMA5 Axis

Chuanjing Zhang^1†^, Yongfei Song^1†^, Xiaojun Jin^1^, Qingbo Xu^2^, Honghua Ye^1^, Zhuonan Wu^3^, Hui Lin^1^, Jiale Hu^1^, Chen Huang^4,5*^, Jianqing Zhou^1*^, Jiangfang Lian^1*^.

Table S1. The daily records of stressors for this study. ^a^

|  | Monday | Tuesday | Wednesday | Thursday | Friday | Saturday | Sunday |
| --- | --- | --- | --- | --- | --- | --- | --- |
| Week2 | 10 | 5 | 8 | 3 | 9 | 2 | 10 |
| Week3 | 9 | 2 | 3 | 6 | 10 | 5 | 2 |
| Week4 | 7 | 8 | 4 | 9 | 5 | 1 | 8 |
| Week5 | 6 | 7 | 1 | 2 | 8 | 7 | 4 |
| Week6 | 6 | 8 | 10 | 9 | 5 | 4 | 2 |
| Week7 | 9 | 3 | 10 | 6 | 7 | 4 | 1 |
| Week8 | 7 | 6 | 9 | 3 | 5 | 1 | 4 |
| Week9 | 6 | 2 | 3 | 5 | 2 | 4 | 10 |
| Week10 | 9 | 8 | 2 | 6 | 3 | 8 | 9 |
| Week11 | 10 | 3 | 7 | 2 | 3 | 10 | 4 |
| Week12 | 2 | 9 | 3 | 5 | 8 | 6 | 4 |
| Week13 | 3 | 9 | 5 | 10 | 5 | 9 | 6 |
| Week14 | 4 | 1 | 8 | 9 | 1 | 3 | 4 |
| Week15 | 5 | 9 | 6 | 5 | 10 | 7 | 10 |
| Week16 | 5 | 6 | 3 | 8 | 1 | 7 | 8 |
| Week17 | 2 | 3 | 9 | 6 | 3 | 5 | 2 |
| Week18 | 1 | 7 | 9 | 8 | 6 | 3 | 2 |
| Week19 | 7 | 1 | 5 | 9 | 5 | 3 | 1 |
| Week20 | 5 | 8 | 4 | 10 | 7 | 6 | 2 |
| Week21 | 8 | 3 | 5 | 4 | 6 | 1 | 4 |

^a^ Stressors are replaced in numbers of 1-10, as follows: 1, 18 hours of food deprivation; 2, 18 hours of water deprivation; 3, 24 hours of wet bedding; 4, 24 hours without bedding material; 5, 24 hours with a 45° cage tilt; 6, 5 minutes of tail clipping; 7, 24 hours of inverted photoperiod; 8, 36 hours of continuous lighting; 9, 30 minutes of cat meow stimulation (60dB); 10, bind for 1h.

Table S2. SiRNA sequences used in this study.

| siRNA | Sense (5’-3’) | Antisense (5’-3’) |
| --- | --- | --- |
| negative control | UUCUCCGAACGUGUCACGUTT | ACGUGACACGUUCGGAGAATT |
| siRNA1 (*GR*) | CUCCUGAUCUGAUUAUUAATT | UUAAUAAUCAGAUCAGGAGTT |
| siRNA2 (*GR*) | GGUGUUGUAUGCAGGAUAUTT | AUAUCCUGCAUACAACACCTT |
| siRNA3 (*GR*) | GGACACGAAUGAGGAUUGUTT | ACAAUCCUCAUUCGUGUCCTT |
| siRNA4 (*GR*) | GCAGAGGAUUCUCCUUGAUTT | AUCAAGGAGAAUCCUCUGCTT |
| siRNA5 (*Lama5*) | GACGGCUGCAUCUUAAGAATT | UUCUUAAGAUGCAGCCGUCTT |
| siRNA6 (*Lama5*) | GGGCACAGAAAUUGUUGGATT | UCCAACAAUUUCUGUGCCCTT |
| siRNA7 (*Lama5*) | GGAUUCUACGGACCUAGCUTT | AGCUAGGUCCGUAGAAUCCTT |
| siRNA8 (*Lama5*) | GUUUCUGCAAGGCUCAUGUTT | ACAUGAGCCUUGCAGAAACTT |


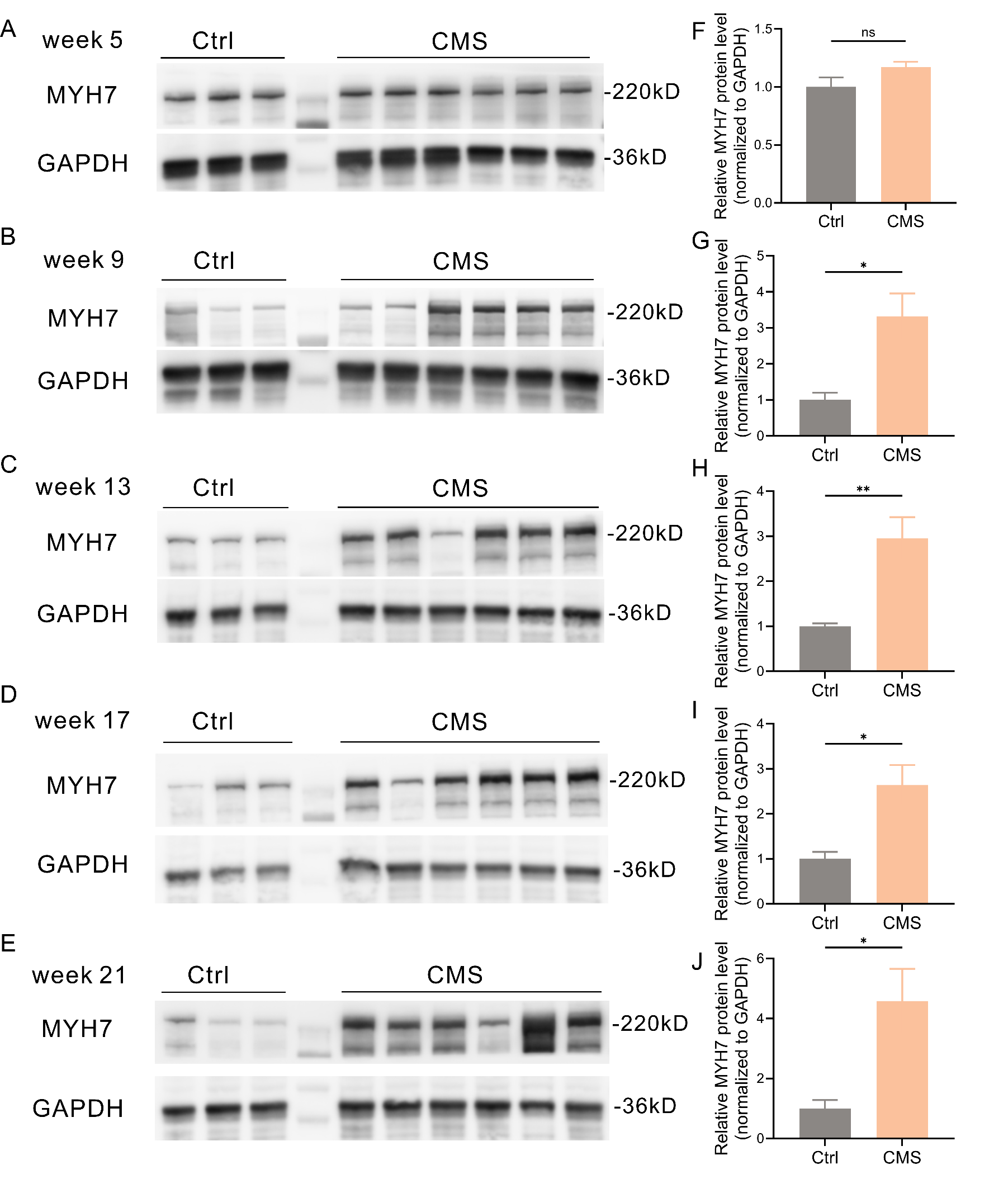


Figure S1. CMS induces increased expression of MYH7 protein in the hearts of rats. Panels (A-E) show representative Western blot images of MYH7 protein in cardiac tissues of control and CMS rats at weeks 5 (A), 9 (B), 13 (C), 17 (D), and 21 (E). Panels (F-J) display quantitative analysis of MYH7 protein expression in cardiac tissues of control and CMS rats at weeks 5 (F), 9 (G), 13 (H), 17 (I), and 21 (J) (n = 3-6). CMS, chronic mild stress; MYH7, myosin heavy chain 7.


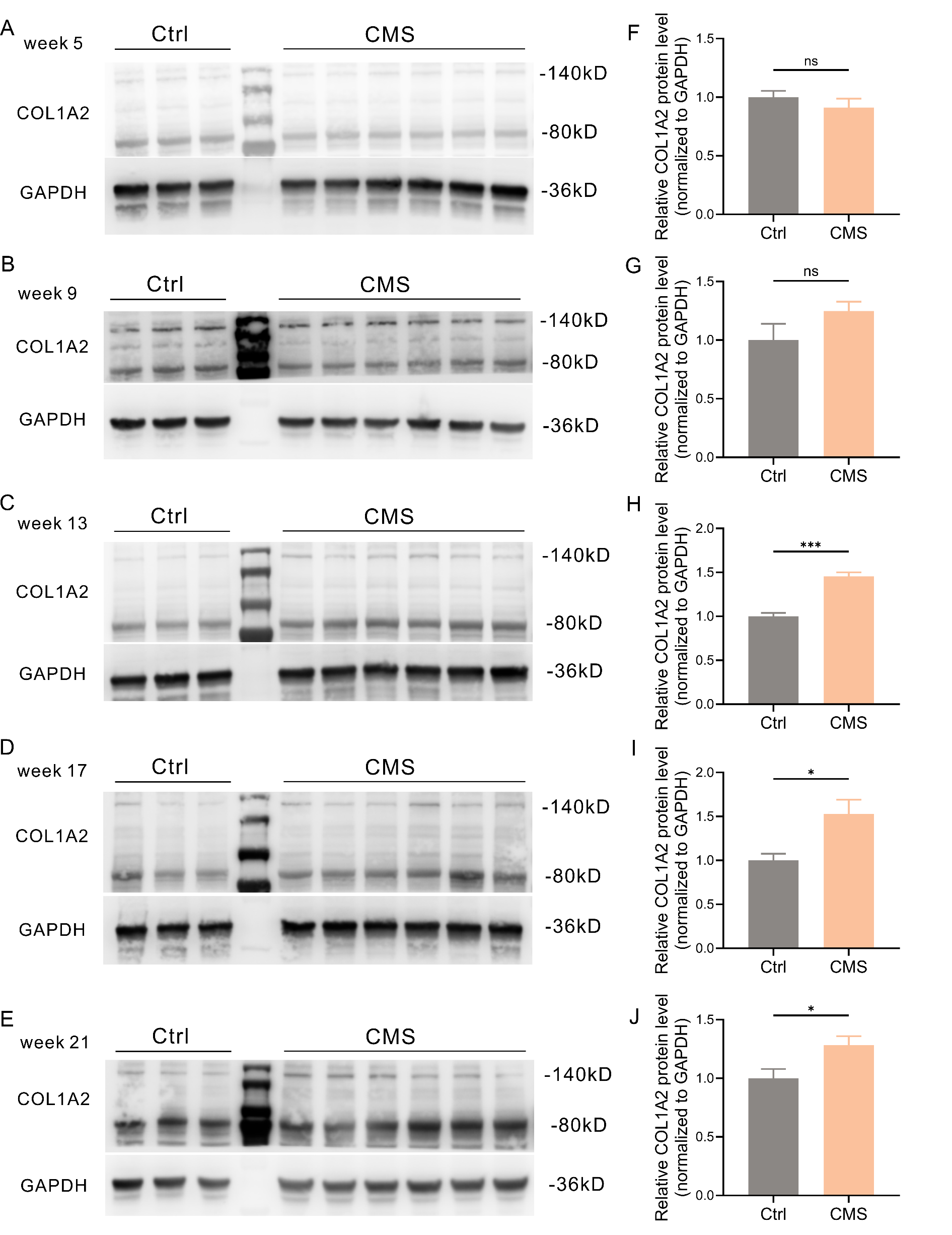


Figure S2. CMS induces increased expression of COL1A2 protein in the hearts of rats. Panels (A-E) show representative Western blot images of COL1A2 protein in cardiac tissues of control and CMS rats at weeks 5 (A), 9 (B), 13 (C), 17 (D), and 21 (E). Panels (F-J) display quantitative analysis of COL1A2 protein expression in cardiac tissues of control and CMS rats at weeks 5 (F), 9 (G), 13 (H), 17 (I), and 21 (J) (n = 3-6). CMS, chronic mild stress; COL1A2, collagen type I alpha 2 chain.


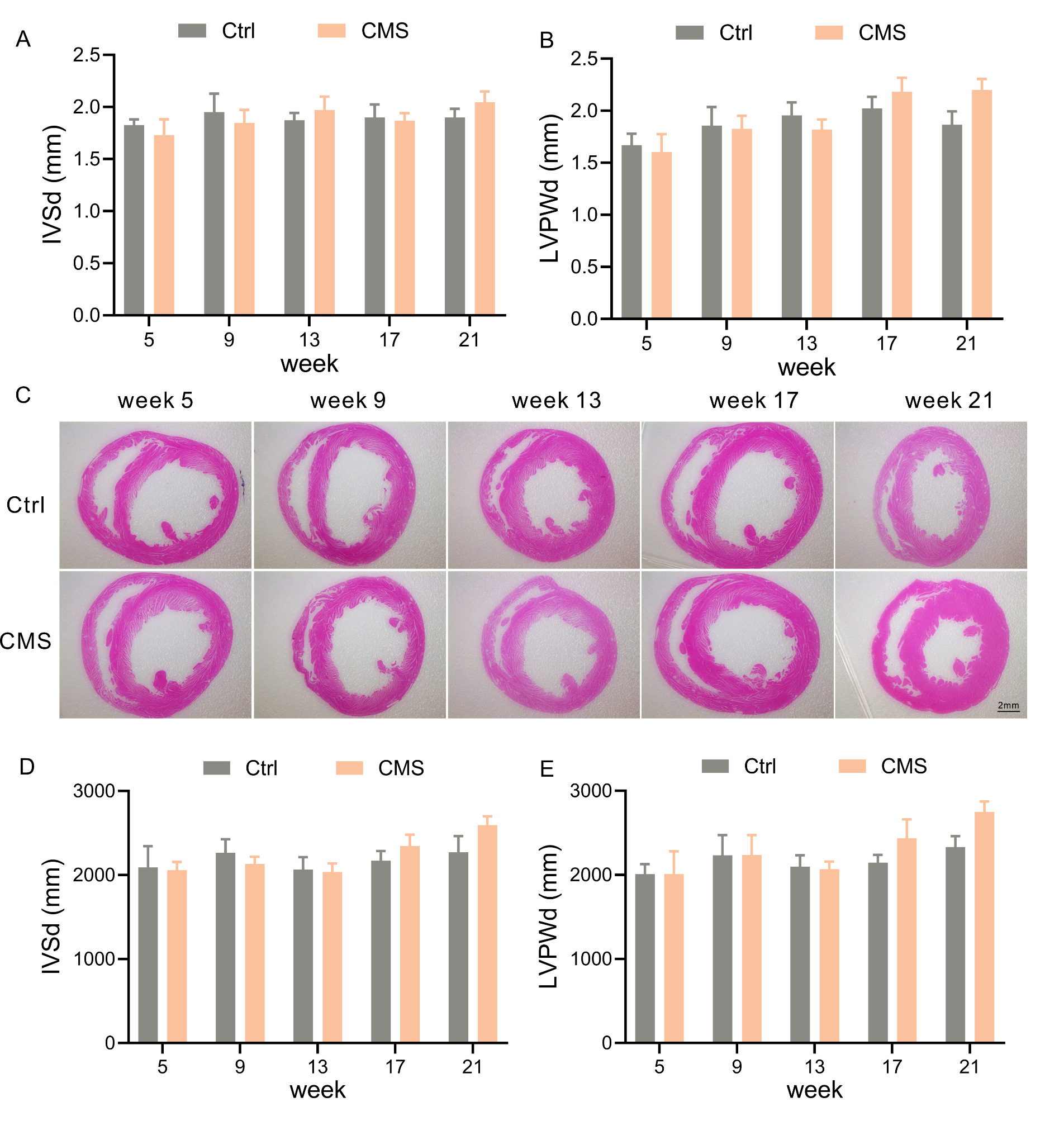


Figure S3. CMS rats show an increasing trend in ventricular thickness. Panels (A-B) display bar graphs of IVSd (A) and LVPWd (B) from echocardiograms of control and CMS rats in five batches (n=4-10). Panel (C) shows representative images of HE staining from cardiac tissues of control and CMS rats in five batches. Panels (D-E) present quantitative analysis of IVSd (D) and LVPWd (E) from HE staining of control and CMS rats in five batches (n=3-7). CMS, chronic mild stress; IVSd, diastolic interventricular septal; LVPWd, diastolic left ventricular posterior wall; HE, hematoxylin eosin.


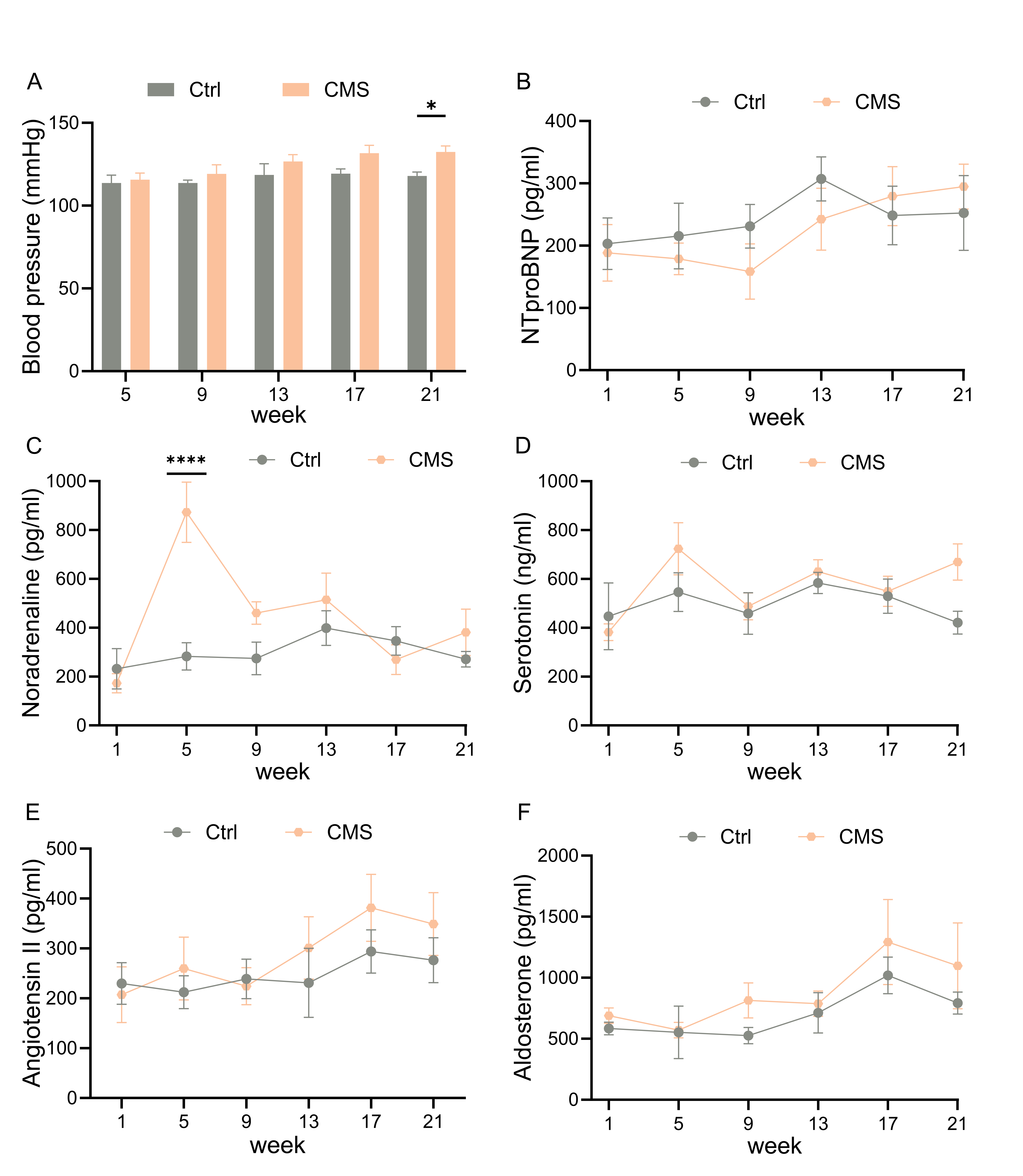


Figure S4. Blood pressure and levels of various serum hormones in control and CMS rats. (A) Trend of systolic blood pressure changes in control and CMS rats in five batches. (B-F) Trend of serum NTproBNP (B), norepinephrine (C), serotonin (D), angiotensin II (E), and aldosterone (F) levels in control and CMS rats in five batches. CMS, chronic mild stress; NTproBNP, N-terminal pro-B-type natriuretic peptide.


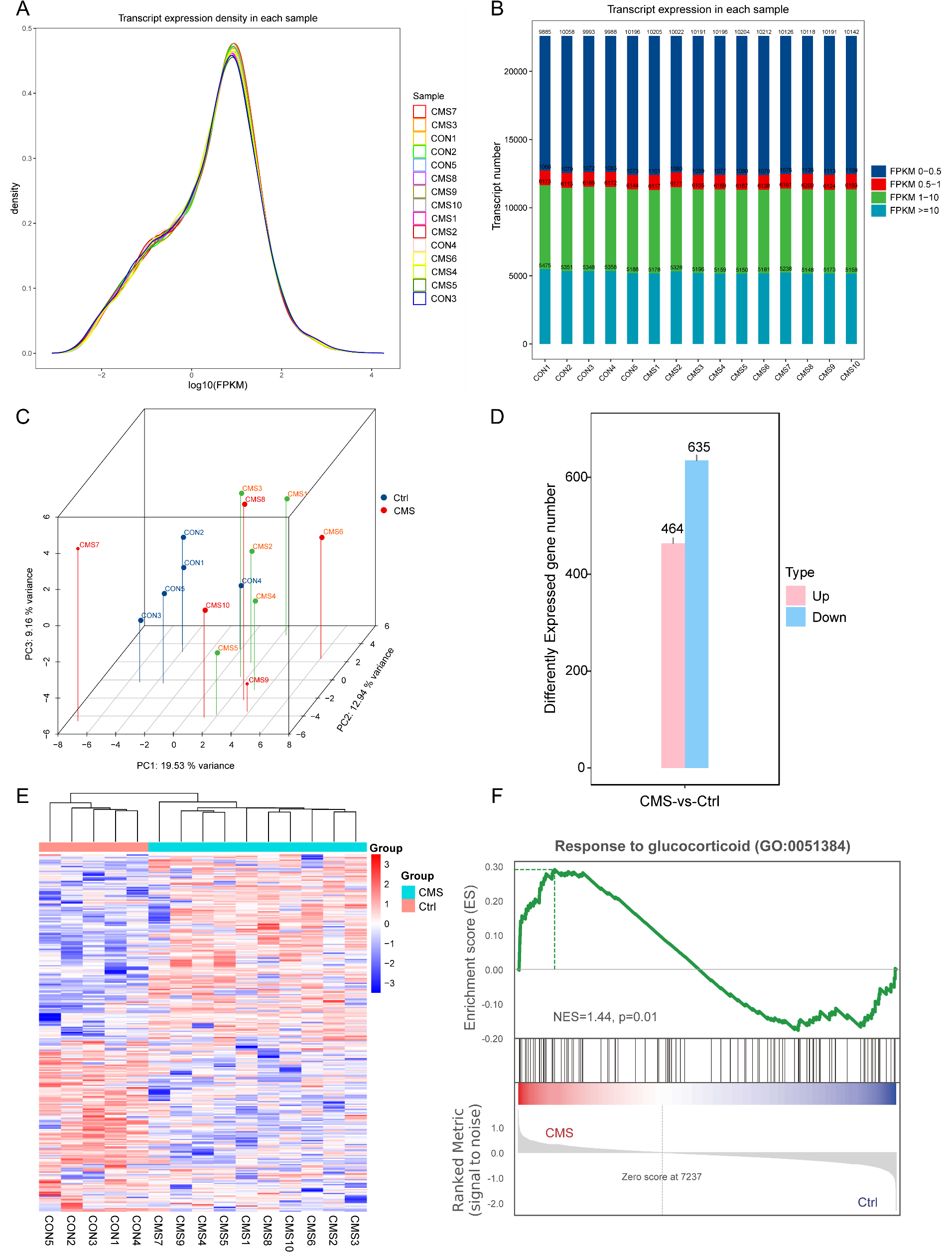


Figure S5. Differential analysis of mRNA sequencing in rat heart tissues. (A-B) Density plot (A) and bar plot (B) showing mRNA transcript expression abundance in samples from control and CMS rats. (C) 3D PCA analysis of samples from control and CMS rats. (D) Number of upregulated and downregulated genes in the hearts of CMS compared to control rats. (E) Heatmap displaying the top 30 differentially expressed genes in the hearts of CMS and control rats. (F) GSEA image of the term "response to glucocorticoid". CMS, chronic mild stress; PCA, principal component analysis; GSEA, Gene Set Enrichment Analysis.


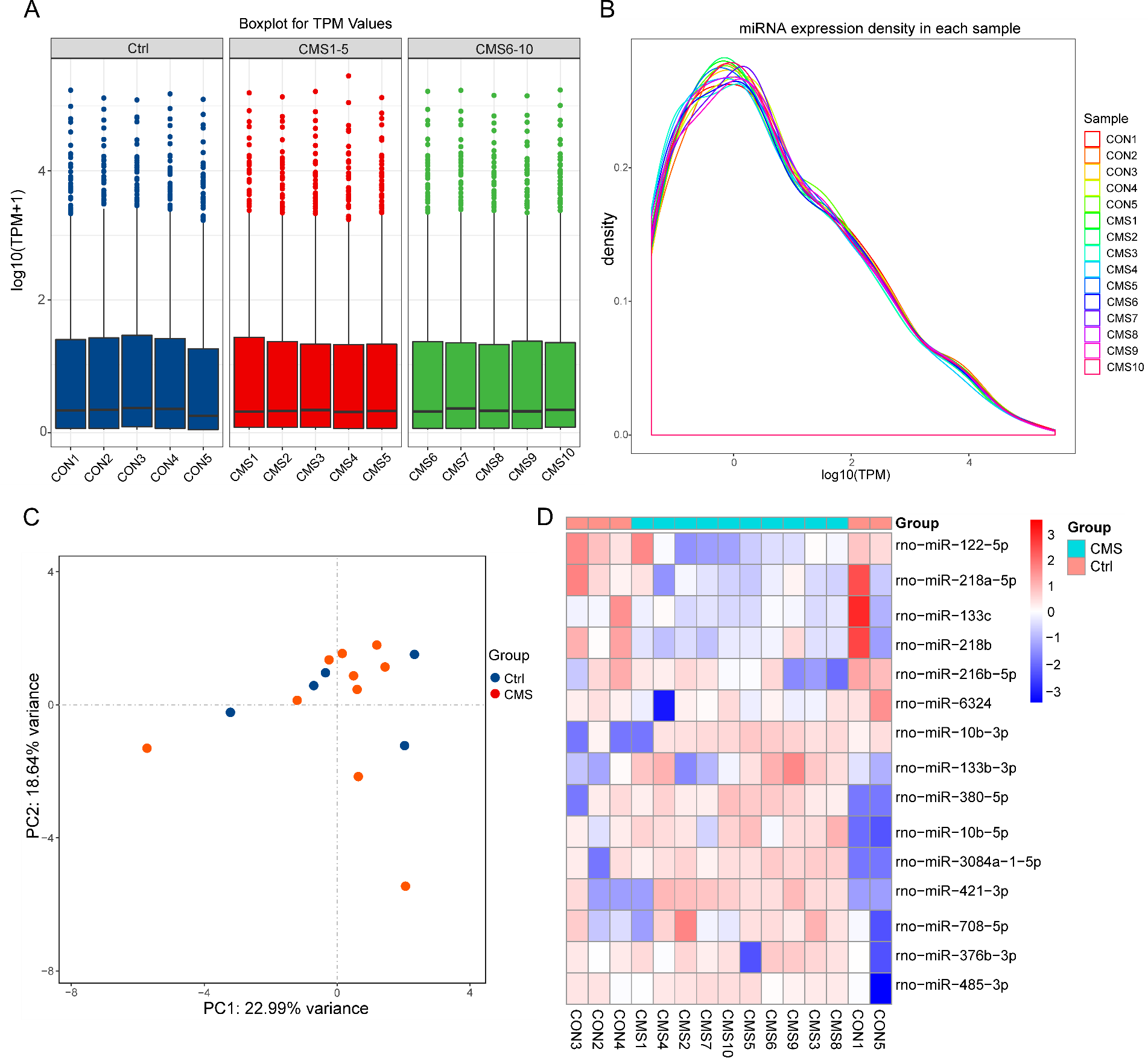


Figure S6. Differential analysis of miRNA sequencing in rat heart tissues. (A) Boxplot (A) and density plot (B) showing the expression abundance of miRNAs in samples from control and CMS rats. (C) PCA plot of miRNAs in heart tissues of control and CMS rats. (D) Heatmap displaying significantly differentially expressed miRNAs in the hearts of CMS and control rats. CMS, chronic mild stress; PCA, principal component analysis.


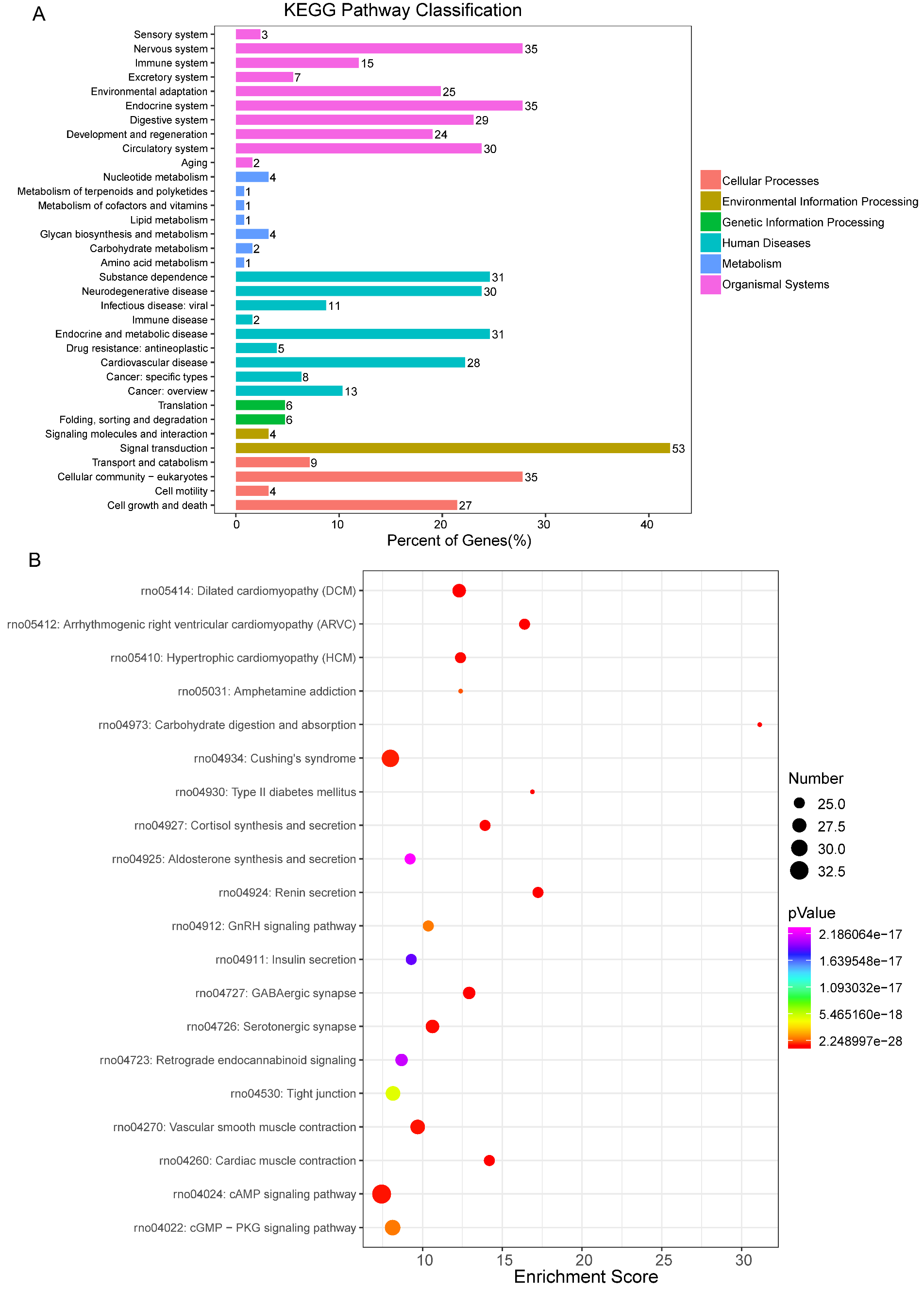


Figure S7. KEGG enrichment analysis of potential target genes of differentially expressed miRNAs in rat heart tissues. (A) Bar plot showing the number of genes involved in KEGG pathway categories of potential target genes of differentially expressed miRNAs. (B) Bubble plot displaying the results of KEGG enrichment analysis for potential target genes of differentially expressed miRNAs. KEGG, Kyoto Encyclopedia of Genes and Genomes.


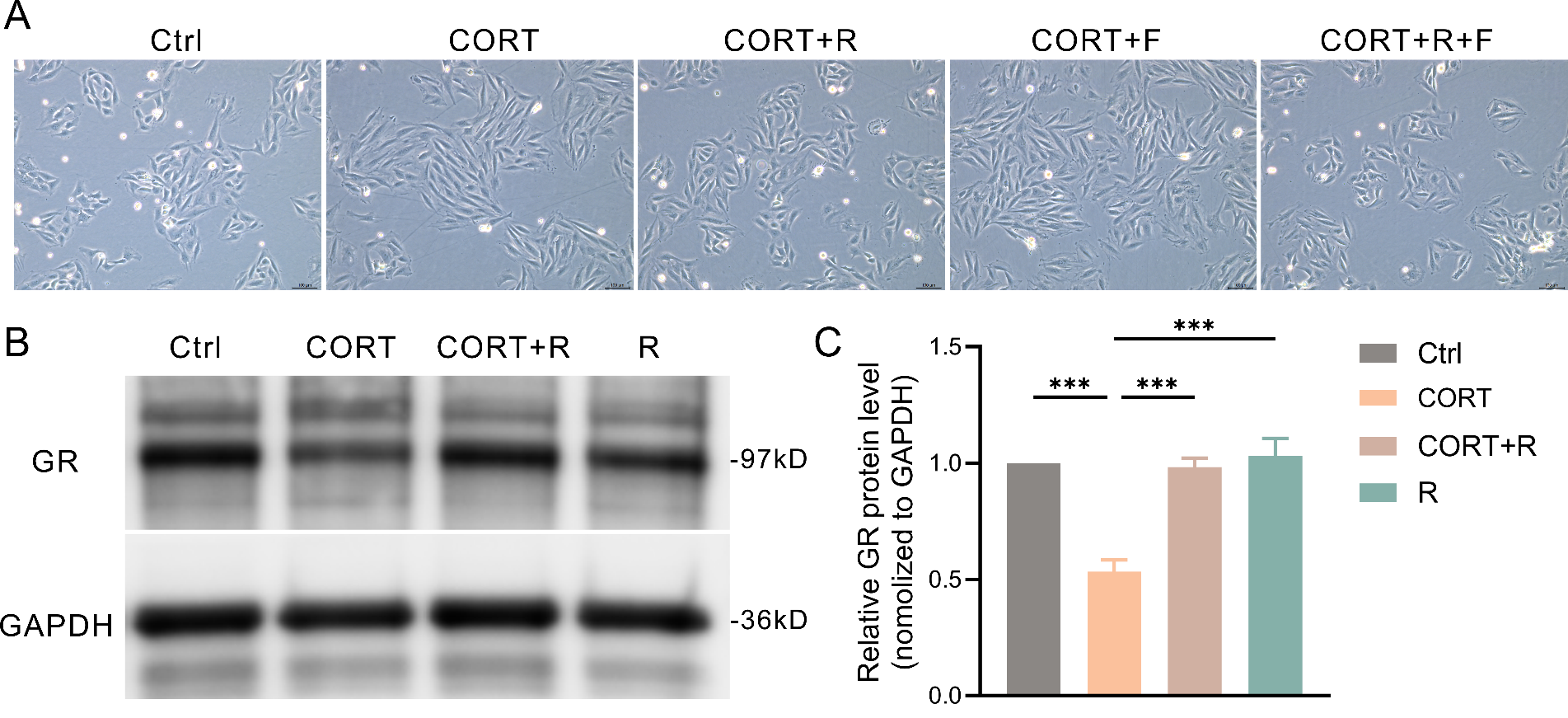


Figure S8. Morphological changes and total GR protein levels of H9C2 cells treated with relacorilant. (A) Representative images of cell morphology in different groups of H9C2 cells. (B-C) Representative Western blot image (B) and quantification analysis (C) of total GR protein expression (n=3) in different groups of H9C2 cells. GR, glucocorticoid receptor; CORT, corticosterone; R, relacorilant; F, finerenone.


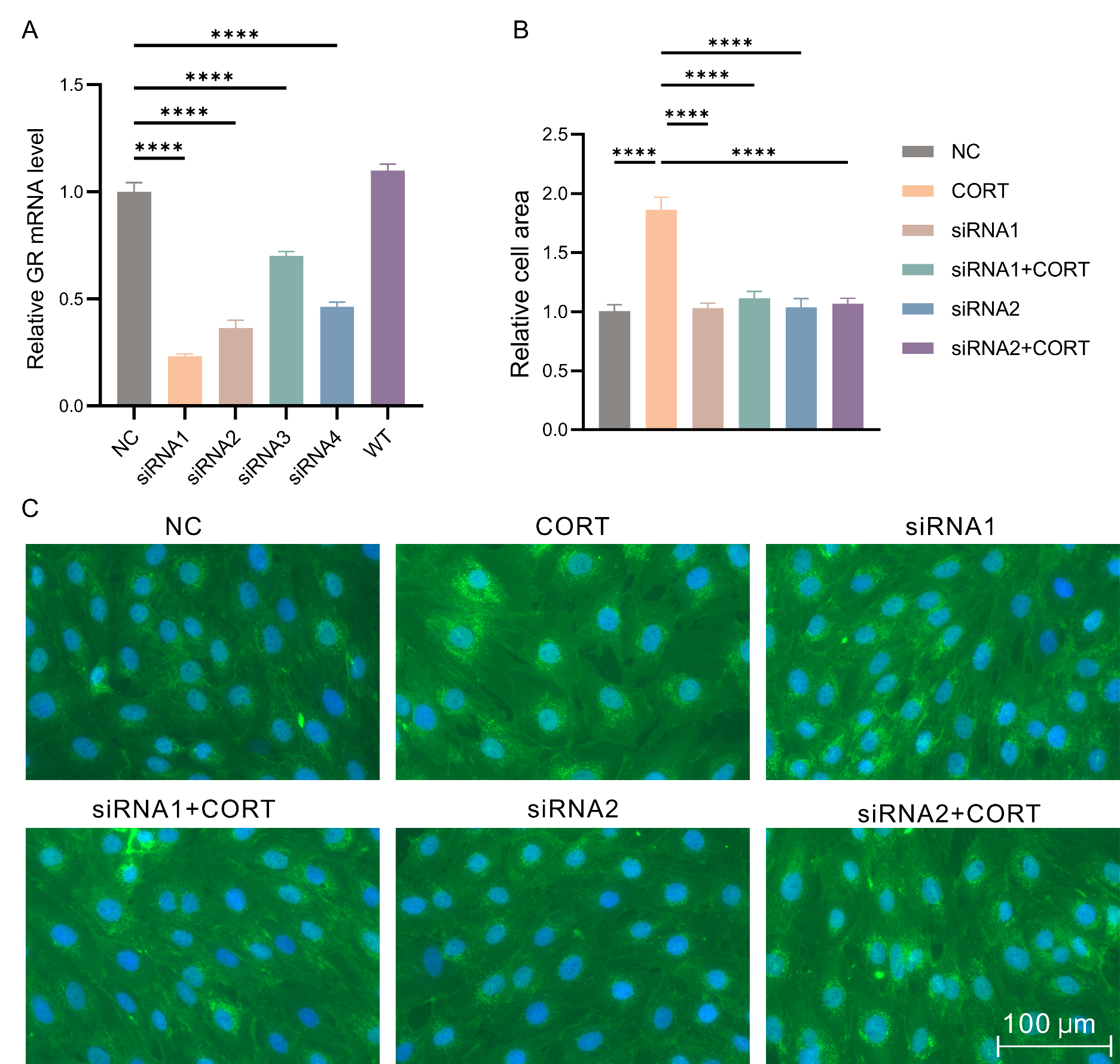


Figure S9. SiRNA screening of GR knockdown and WGA staining. (A) Relative expression levels of GR in H9C2 cells using four siRNAs. (B-C) Quantification analysis (B) and representative images of WGA staining (C) in different groups of H9C2 cells. GR, glucocorticoid receptor; WGA, wheat germ agglutinin.


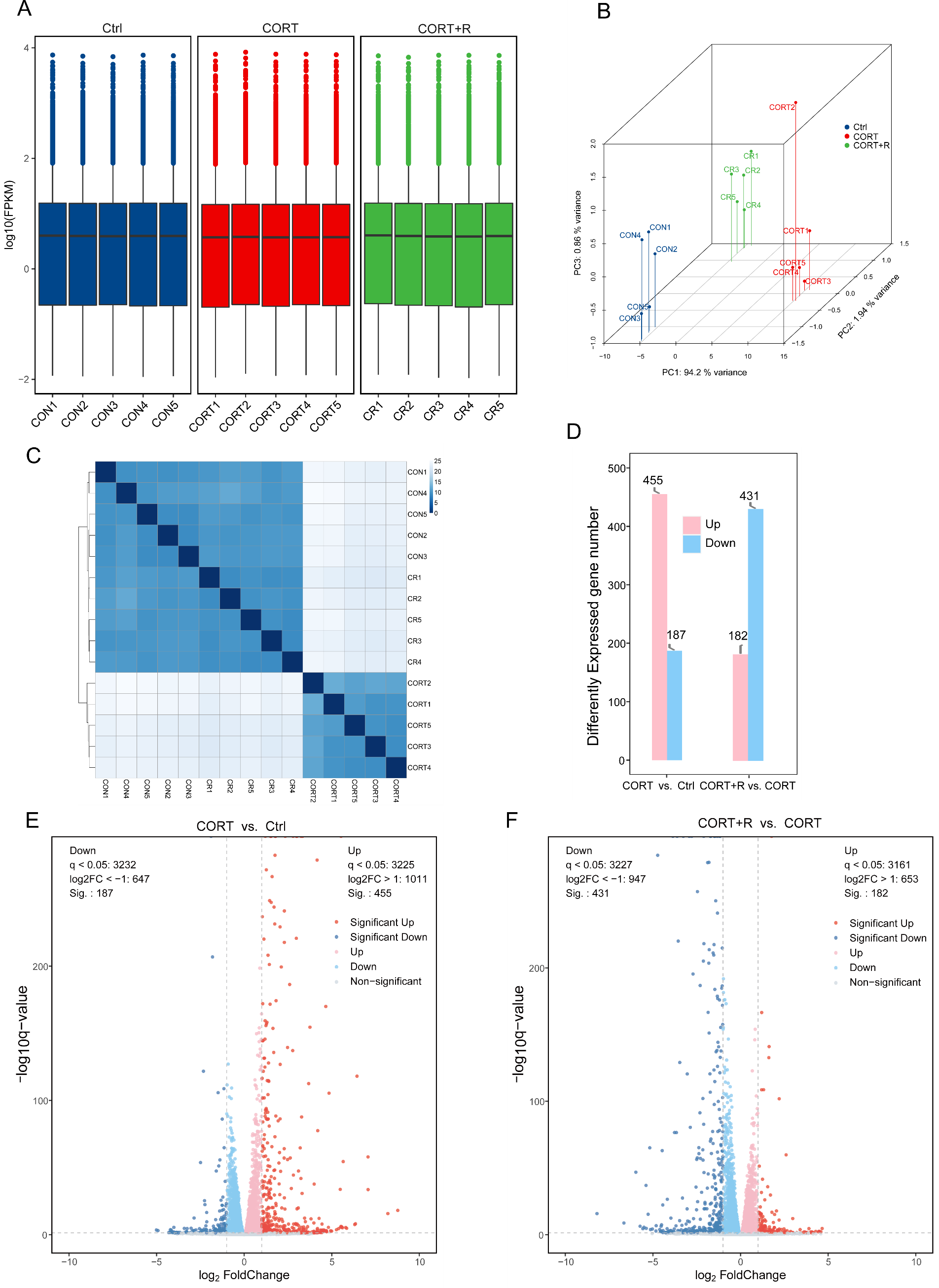


Figure S10. Differential transcriptome analysis in H9C2 cells. (A) Boxplot showing mRNA transcript expression abundance in different groups of H9C2 cells. (B) 3D PCA plot displaying the differential mRNA expression distribution among samples. (C) Cluster heatmap showing the similarity of mRNA expression among samples. (D) Bar plot indicating the number of differentially expressed genes in CORT vs. Ctrl and CORT+R vs. CORT comparisons. (E) Volcano plot illustrating the differentially expressed genes in CORT vs. Ctrl comparison. (F) Volcano plot illustrating the differentially expressed genes in CORT+R vs. CORT comparison. PCA, principal component analysis; CORT, corticosterone; R, relacorilant; F, finerenone.


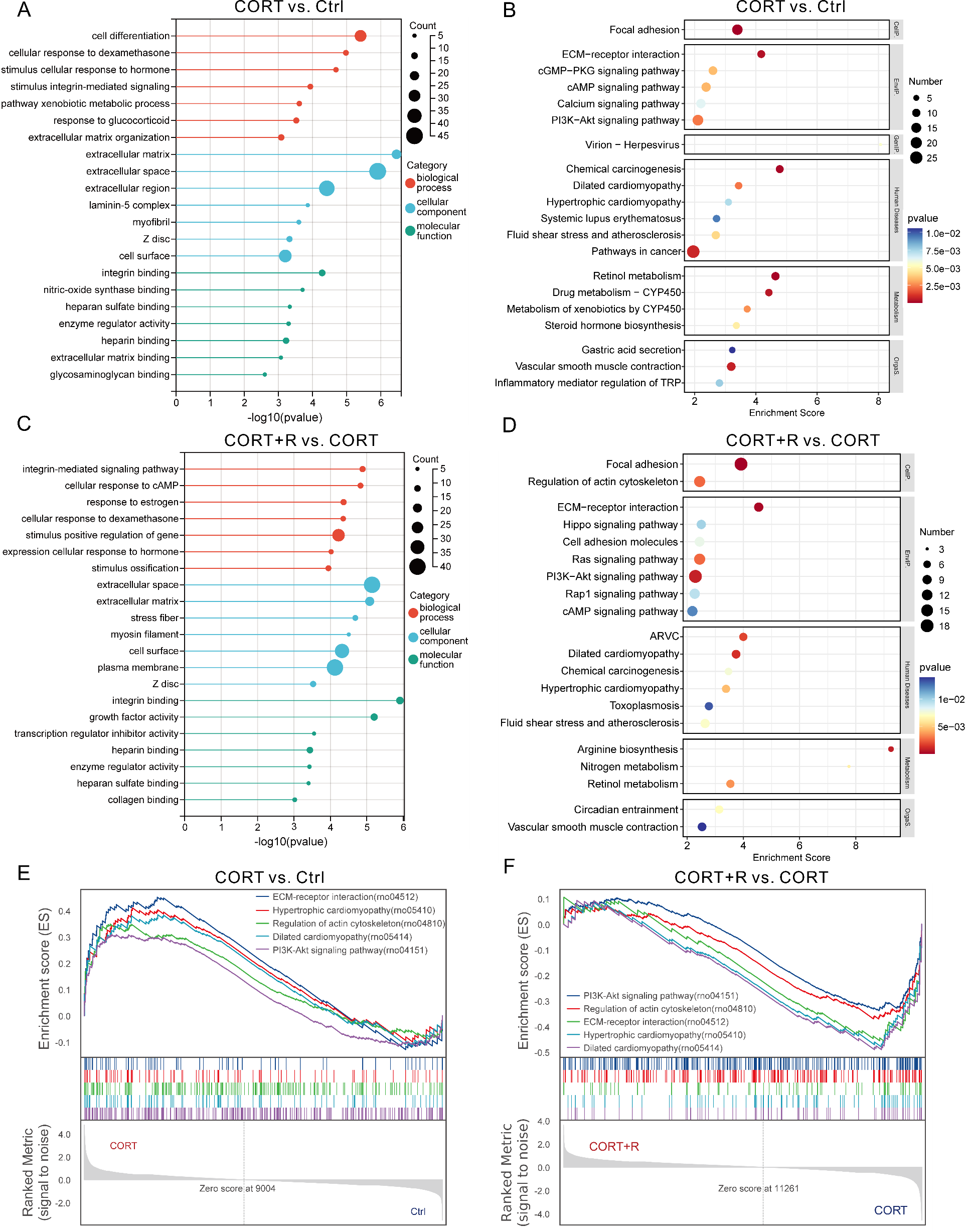


Figure S11. Enrichment analysis of differentially expressed genes in H9C2 cells. (A-B) Major categories of GO enrichment analysis (A) and KEGG enrichment analysis results (B) for differentially expressed genes in CORT vs. Ctrl comparison. (C-D) Major categories of GO enrichment analysis (C) and KEGG enrichment analysis results (D) for differentially expressed genes in CORT+R vs. CORT comparison. (E, F) GSEA results of major hypertrophy related pathways in CORT vs. Ctrl comparison (E) and CORT+R vs. CORT comparison (F). GO, Gene Ontology; KEGG, Kyoto Encyclopedia of Genes and Genomes; GSEA, Gene Set Enrichment Analysis; CORT, corticosterone; R, relacorilant; F, finerenone.


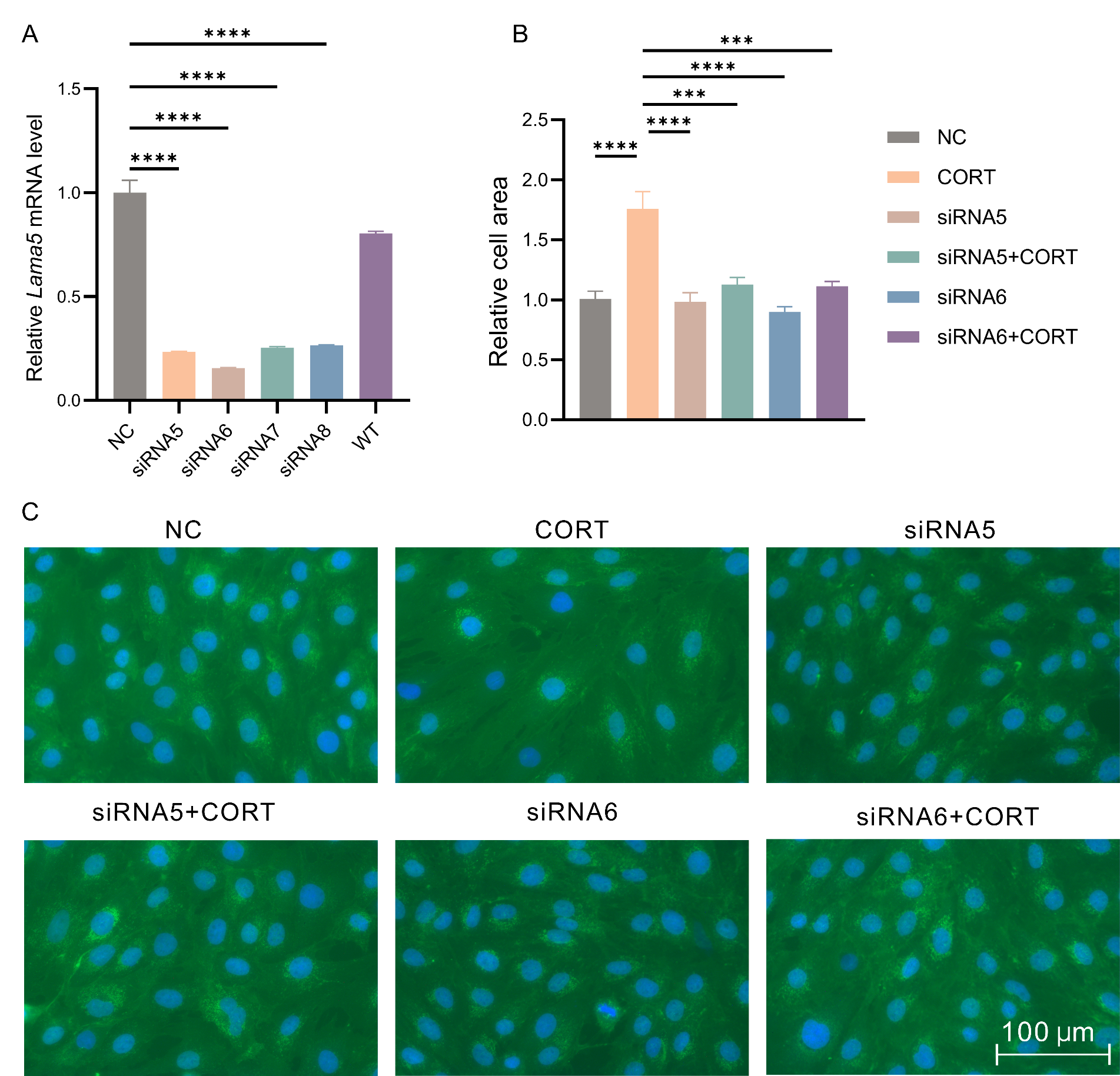


Figure S12. SiRNA screening of *Lama5* knockdown and WGA staining. (A) Relative expression levels of *Lama5* in H9C2 cells using four siRNAs. (B-C) Quantification analysis (B) and representative images of WGA staining (C) in different groups of H9C2 cells. *Lama5*, laminin subunit alpha 5; WGA, wheat germ agglutinin.


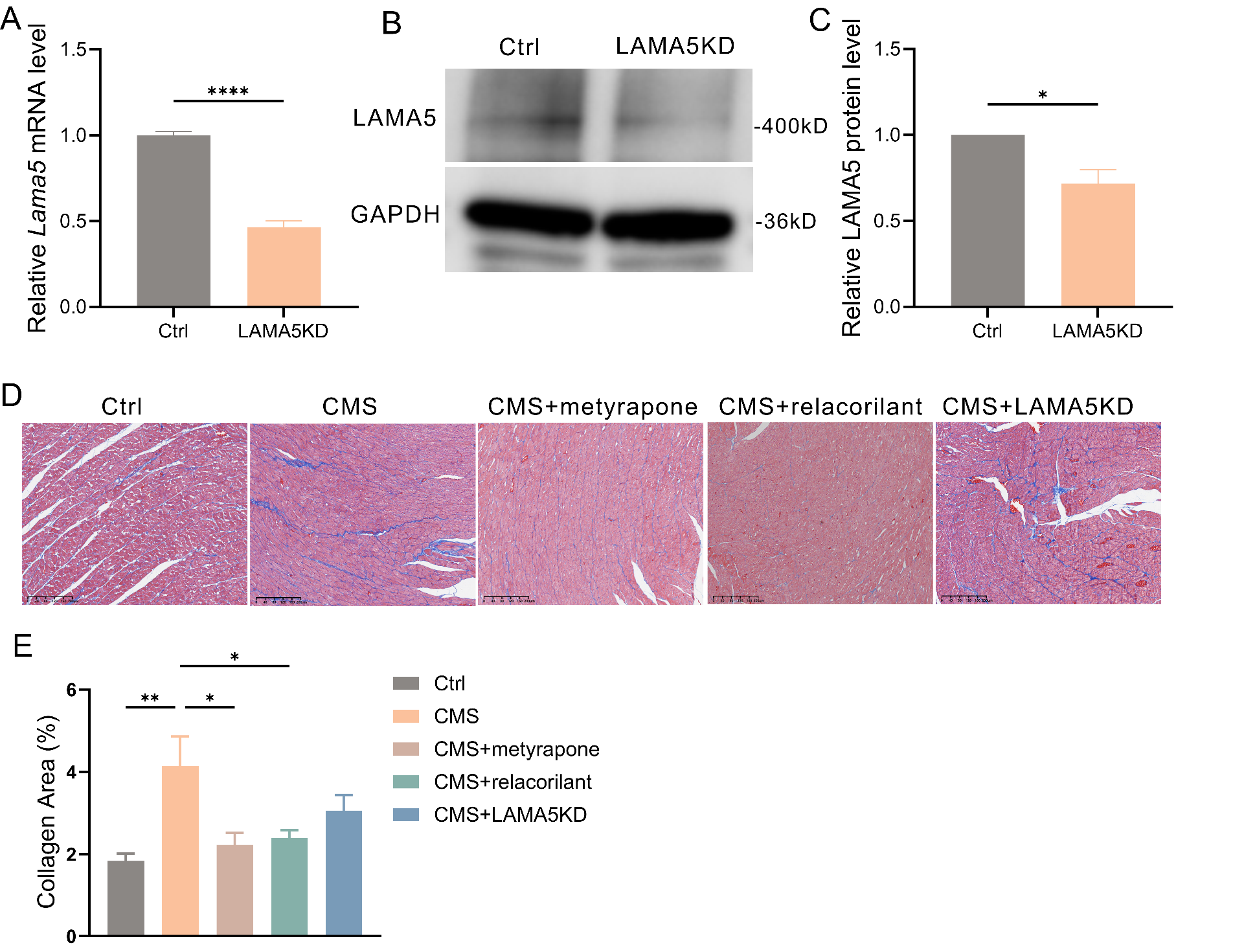


Figure S13. Knockdown efficiency of LAMA5 by AAV and Masson's trichrome staining of heart tissues in different groups of rats. (A) Relative mRNA expression levels of LAMA5 in the hearts of LAMA5KD and control rats (A), representative Western blot images of LAMA5 protein (B), and quantitative analysis (C). (D-E) Representative images of Masson trichrome staining in heart tissue sections of rats in different groups (D) and quantitative analysis (E). LAMA5, laminin subunit alpha 5; AAV, adeno-associated virus.


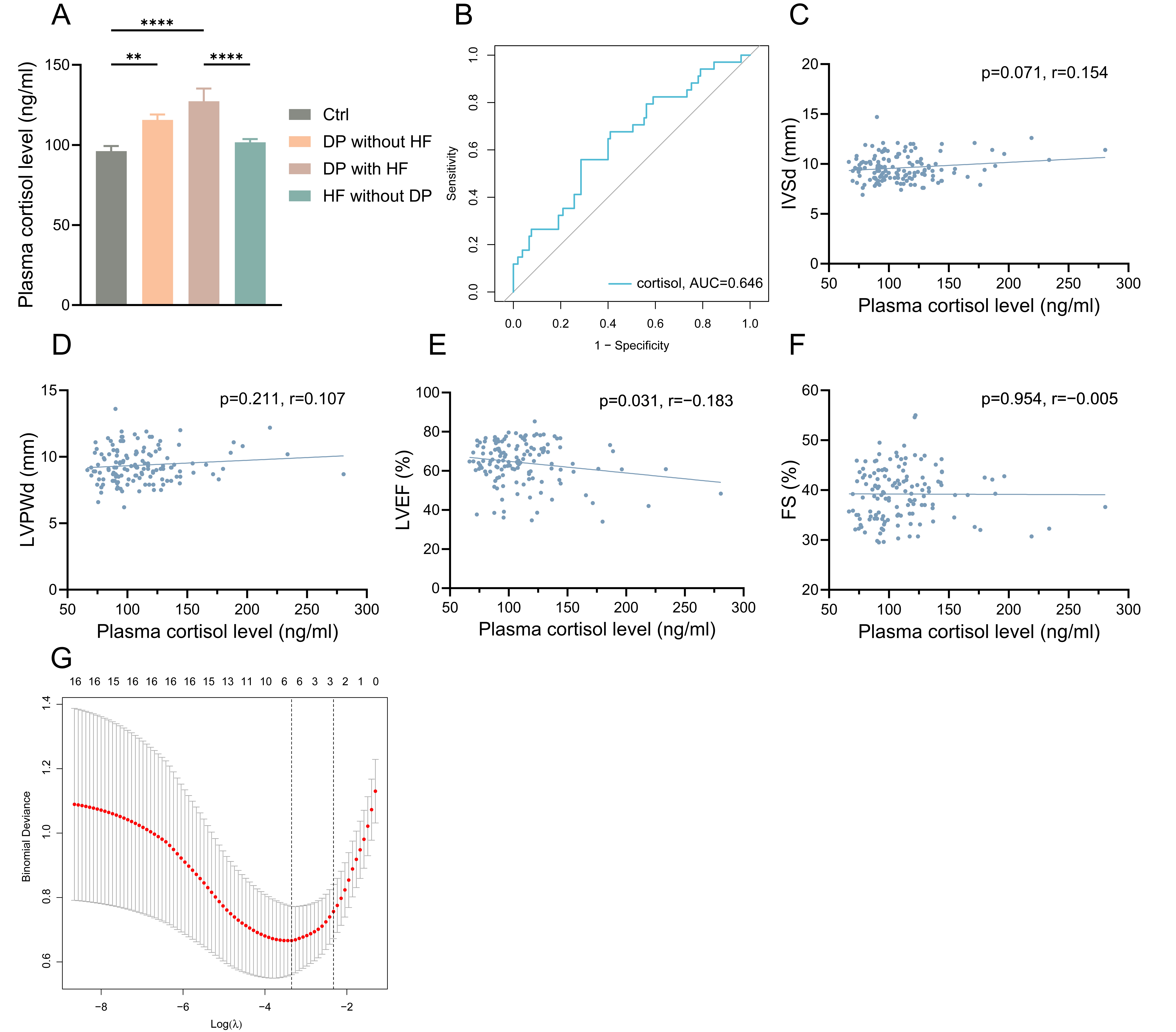


Figure S14. Plasma corticosterone levels and LASSO regression for diagnostic biomarker screening in clinical samples. (A) Bar graph displaying plasma corticosterone levels in different groups of clinical samples. (B) ROC analysis showing the diagnostic value of plasma corticosterone levels for DP with HF. (C-F) Scatter plots depicting the correlation between plasma corticosterone levels and IVSd (C), LVPWd (D), LVEF (E), and FS (F). (G) LASSO regression for screening diagnostic biomarkers of DP with HF based on multiple clinical indicators, resulting in a minimum of two biomarkers. LASSO, least absolute shrinkage and selection operator; ROC, receiver operation characteristic; DP, depression; HF, heart failure; IVSd, diastolic interventricular septal; LVPWd, diastolic left ventricular posterior wall; LVEF, left ventricle ejection fraction; FS, fractional shortening.
